# Machine learning recovers folk classification of *Banisteriopsis caapi* from herbarium leaves an ayahuasca liana

**DOI:** 10.1016/j.isci.2026.115753

**Published:** 2026-04-15

**Authors:** Scheila Cristina Biazatti, Deborah Bambil, Rômulo Môra, Lúcio Flávio de Alencar Figueiredo, Regina Célia de Oliveira

**Affiliations:** 1 Department of Botany, Biology Institute, University of Brasília (UnB), Brasília, DF 70910-900, Brazil; 2 Department of Forest Engineering, Federal University of Rondônia (UNIR), Rolim de Moura, RO 76940-000, Brazil; 3 Embrapa Genetic Resources and Biotechnology/National Institute of Science and Technology in Synthetic Biology, Brasília, DF 70770-917, Brazil; 4 Department of Forest Science, Federal Rural University of Pernambuco (UFRPE), Recife, PE 52171-900, Brazil

**Keywords:** Biological sciences

## Abstract

Ayahuasca refers to an entheogenic brew and its main vine, *Banisteriopsis caapi*, whose high morphological diversity underlies traditional folk classifications. This study evaluated whether machine learning algorithms can recover folk classifications using images of dried leaves. We analyzed 47 vine plants, mainly *B. caapi* folk types and *Diplopterys cabrerana* as an outgroup, using adaxial and abaxial leaf image features related to color, shape, texture, and filters. All evaluated algorithms showed statistically similar performance; however, support vector machine (SVM) achieved the highest accuracy, reaching 70% overall and over 90% for the folk types *Hybrid* and *Arara*, and the related outgroup species *D. cabrerana*. Lower accuracy in *Cabi* and *Quebrador* reflects morphological overlap. Confusion matrix and similarity network analyses showed only partial agreement with previous ethnobotanical classifications. Focusing on subtle variation within a single species, this study demonstrates that integrating traditional knowledge with machine learning enables automated validation of folk taxonomies.

## Highlights

• Machine learning recovers folk classification of *Banisteriopsis caapi*
• Leaf morphology contains signals of traditional ethnobotanical taxonomy
• Combined adaxial and abaxial leaf images improve classification accuracy
• AI tools can support taxonomy of culturally important plant species

## Graphical abstract

**Figure:**
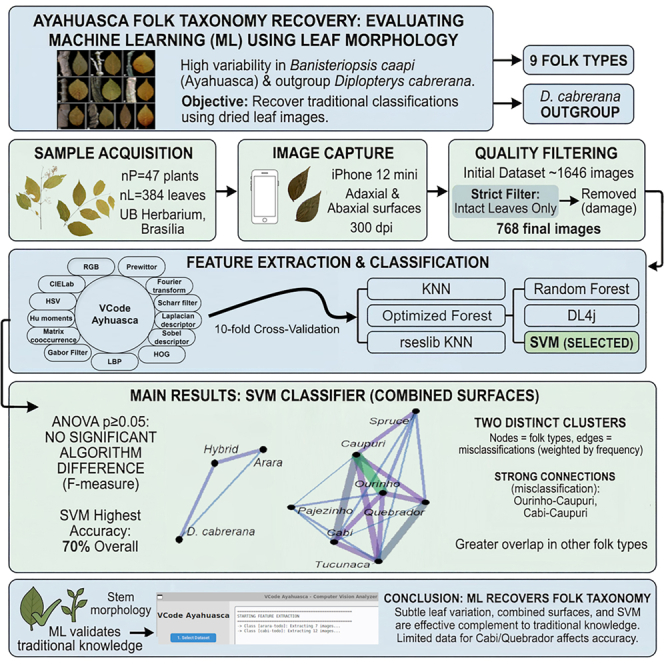

## Introduction

*Ayahuasca* is a term that refers both to a ritualistic brew and to the vine *Banisteriopsis caapi* Spruce (ex Griseb.) C.V. Morton (Malpighiaceae) and some others Malpighiaceae species, component of this traditional Amazonian beverage.^1^^,^^2^^,^^3^^,^^4^ The most widespread composition of the ayahuasca brew results from the decoction of stems (and/or roots) of *B. caapi*, which contains the β-carbolines harmine, harmaline, and tetrahydroharmine, joined with the leaves of *Psychotria viridis* Ruiz & Pav. (Rubiaceae), which contains N,N-dimethyltryptamine (DMT), the compound responsible for the brew’s perceptual and visionary effect.^5^^,^^6^ In order for DMT to produce effects when taken orally, monoamine oxidase (MAO) must be inhibited, an action provided by the β-carbolines present in the vine.^6^

The historical record of ayahuasca use extends throughout the northwestern Amazon, encompassing 33 ethnic groups across Brazil, Bolivia, Colombia, Ecuador, and Peru, as well as the Orinoco region in Venezuela and the Pacific coast of Colombia and Panama.^7^^,^^8^^,^^9^ Today, ayahuasca use has spread significantly, reaching diverse cultural and geographic contexts in several countries across Europe, Asia, Oceania, and the Americas.^10^^,^^11^ In Brazil, its use initially expanded through syncretic Christian religions, mainly Santo Daime, Barquinha, and União do Vegetal, which later influenced other urban practices of religious, therapeutic, and recreational nature, also reaching non-Amazonian Indigenous groups.^12^ The growing use of ayahuasca has led Peruvian shamans to denounce the intense “spiritual extractivism” of the plants used in the brew.^13^

The recognition of different types of *B. caapi* by traditional peoples has been documented by several authors.^4^^,^^7^^,^^14^ Brazil’s syncretic religious groups recognize many folk *B. caapi* types.^4^ This folk classification is based primarily on stem morphology, such as the presence or absence of swollen nodes, the formation or absence of braids, the ease or difficulty and resulting shape of stem fiber pounding, and the color of the inner bark. However, the characteristics of the brews, such as taste, density, coloration, and cognitive effects, are predominant criteria.^4^

Initially, only two folk types (*Caupuri* and *Tucunaca*) of *B. caapi* by the Brazilian religious groups ayahuasca were registered.^6^ However, a larger number of other folk types (*Caupuri*, *Tucunaca*, *Arara*, *Pajezinho*, *Ourinho*, among others) were later included based on broader sampling.^4^^,^^15^^,^^16^

This taxonomic difficulty is further compounded by the fact that ayahuasca is not prepared from a single botanical species, but rather from a complex of closely related taxa within Malpighiaceae.^4^^,^^7^^,^^17^^,^^18^ In addition to *B. caapi*, some species of *Banisteriopsis*, *Diplopterys*, and *Tetrapterys* have been reported as components of the brew, whose delimitation has long been controversial and historically unstable. The recognition of these taxa often relies on stem and floral characters, which are rarely preserved in vine and woody herbarium material, blurring the boundaries between folk categories, botanical species, and chemically distinct lineages.^4^^,^^15^

Record of these folk types in herbaria are limited, and many available vouchers are sterile specimens, i.e., composed only of branches and leaves, hinders proper botanical identification, since the taxonomy of *Banisteriopsis* has traditionally been based on the morphology of flowers and fruits.^18^^,^^19^^,^^20^ Herbarium specimens of woody plants conventionally lack the main stem, making it impossible to consider folk classification.^4^ Eight lineages (or lines) of *B. caapi* were identified using DNA barcoding,^16^ but no morphological traits were found to distinguish them as taxonomic entities, and the collectors did not prepare herbarium vouchers for morphological studies.

In botany, species have traditionally been based on morphological characteristics, which align with the classical Linnaean approach. Even among animals, such as bees, species are described as the basic unit used to understand evolutionary biology and biodiversity, playing a key role in conservation and the formulation of public policies.^21^ However, widely used concepts of species, such as morphological, biological, and phylogenetic, face limitations when applied broadly due to hybridization zones, gene flow, and continuous morphological variation.^22^ Thus, in botany, species are often understood as groups of individuals with relative morphological and genetic stability, capable of maintaining their identity even in contexts of ecological contact and potential gene flow with related taxa.^22^^,^^23^^,^^24^

In this context, species identification, generally performed by taxonomic specialists, can be a time-consuming and error-prone process, especially in groups with high diversity and overlapping morphological characters. To make this process more agile and accurate, automated identification techniques have been successfully tested, particularly through leaf image analysis. However, studies conducted so far have focused mainly on genetically distant taxa, belonging to different genera and families, achieving classification accuracy rates above 90%.^25^^,^^26^^,^^27^^,^^28^^,^^29^

In recent years, machine learning and artificial intelligence have been increasingly applied to plant systematics and automated species identification, particularly leaf images and morphometric descriptors. Leaves are the most readily available organs throughout a plant’s life. Studies on diverse plant groups have demonstrated the potential of these approaches to detect subtle morphological patterns and improve classification accuracy.^25^^,^^27^^,^^30^

A little before machine learning stabilized for plant classification, infrared (IR) spectroscopy had already achieved excellent results in plant classification,^31^^,^^32^^,^^33^ serving as a complementary tool for plant taxonomists. But for that, an IR instrument is necessary in an air-conditioned room. Portable IR is more flexible, less expensive and yields results close to those of bench IR equipment for classification analysis^34^; however, bench IR delivers yet better quantitative prediction performances^34^^,^^35^ and is more cumbersome for spectra acquisition on exsiccates than portable IR. Using machine learning, data such as photographic images from ordinary cell phones were used to achieve excellent classification.^25^ For this purpose, it yields the same results with a lower budget and faster turnaround. With IR, it is possible to predict the organic composition using high-quality calibrations,^36^^,^^37^ whereas this is not possible with machine learning using pictures. Still, images associated with data attributes are increasingly used in machine learning.^38^

Lately, optoelectronic measurements coupled with machine learning, including optical imaging and spectroscopy, have emerged as powerful tools for high-precision discrimination of complex biological and chemical samples.^39^^,^^40^ These advances highlight the relevance of computational methods as complementary tools to traditional taxonomy, especially in complex and morphologically overlapping groups.

The high classification capability using algorithms, such as deep learning, random forest, and support vector machine (SVM), to capture complex patterns associated with plant morphological features achieve near perfection.^26^ In addition, they emphasize the usefulness of computer vision in contexts where manual identification is limited by the lack of specialists or by the subjectivity of traditional processes.

Moreover, this pioneering study focuses on genetically closely related organisms, a context that represents a greater methodological challenge compared to previous studies involving distantly related species. The task involves subtle morphological variation within a single species, which tests the limits of current machine learning approaches and provides new perspectives for the identification and conservation of *B. caapi*.

During the data collection that led to the study by Oliveira et al.,^4^ a collection of approximately 200 herbarium vouchers containing specimens of *B. caapi* identified by their respective folk names was assembled. These vouchers are part of the present study and represent an important foundation for analyzing the folk classification of the species through leaf morphology. In addition, there is an ongoing effort to expand this collection through new samplings and identifications, aiming to capture the greatest possible diversity of these taxa, which hold significant cultural and botanical relevance.

In addition to its ethnobotanical relevance, this study also engages a central technical challenge in machine learning for plant identification: how do different classification algorithms perform when applied to a complex taxonomic context involving genetically close folk types? Machine learning models use distinct mathematical strategies to identify patterns in data, and their performance may vary substantially according to the nature of the task. Therefore, it is essential to understand whether certain algorithms perform better than others in the classification of *B. caapi* folk types based on leaf morphology, and whether combining adaxial and abaxial leaf surfaces improves classification accuracy.

In this context, the central research question of this study is whether leaf morphology alone contains sufficient information to recover and discriminate the folk classification of *B. caapi* using machine learning approaches. Based on this framework, we tested the following hypotheses: (1) herborized leaves morphometric traits contain detectable features of folk classification in *B*. *caapi*; (2) machine learning classifiers are able to recover part of the traditional categorization based solely on leaf images; and (3) the recovered patterns reflect continuous intra-specific variation, rather than discrete taxonomic entities.

Thus, this study aimed to evaluate whether machine learning could recover the folk classification based on the morphological characteristics of *B. caapi* folk type leaves. Leaf image-based recognition may significantly impact the taxonomy of the species, offering a complementary tool to traditional taxonomic approaches, contributing to a better understanding of its morphological and genetic diversity, and supporting conservation strategies. Furthermore, this research highlights how the integration of ethnobotanical knowledge and modern computational tools can advance the automated identification of culturally significant plants.

## Results

### Classification algorithm performance and leaf surface influence

**Figure 1:**
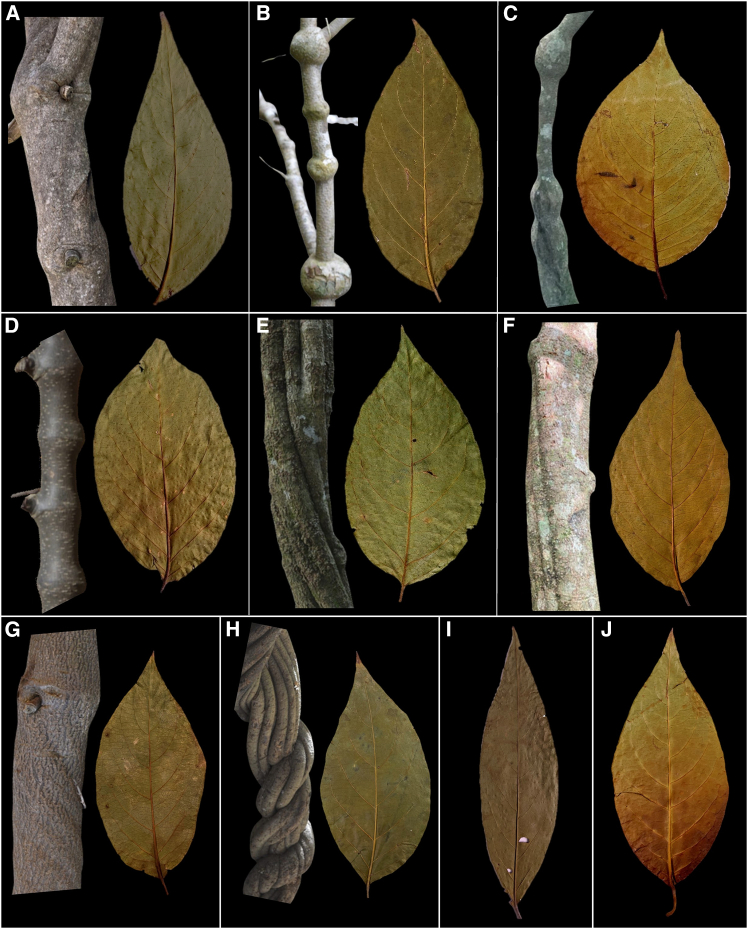
Morphological diversity of *B. caapi* and *D. cabrerana* (A–G) Stem and leaf diversity. (A) *Arara*. (B) *Caupuri*. (C) *Caupuri-de-nós-longos* (*Spruce*). (D) *Hybrid*. (E) *Ourinho*. (F) *Pajezinho*. (G) *Quebrador*. (H) *Tucunaca*. (I and J) Leaves. (I) *D. cabrerana.* (J) *Cabi*.

A total of 768 images of the adaxial and abaxial surfaces of 384 herborized leaves, collected from 47 vine plants of folk types of *B. caapi* (∼95%) and *D. cabrerana* (∼5%) were photographed (*n* = 768), as illustrated in Figure 1, which shows representative images of leaves and stems used in the analyses, and analyzed using six machine learning algorithms (KNN, Rseslib KNN, optimized forest, random forest, SVM, and DL4J) to verify whether the classification assigned based on morphological leaf traits could serve as a potential taxonomic classification tool. The automated identification was performed using the VCodeAyahuasca software, which features an intuitive graphical user interface (GUI) to facilitate use by botanists and researchers. The tool is open-source and available for the scientific community at: https://github.com/DeborahBambil/VCodeAyahuasca.

**Figure 2:**
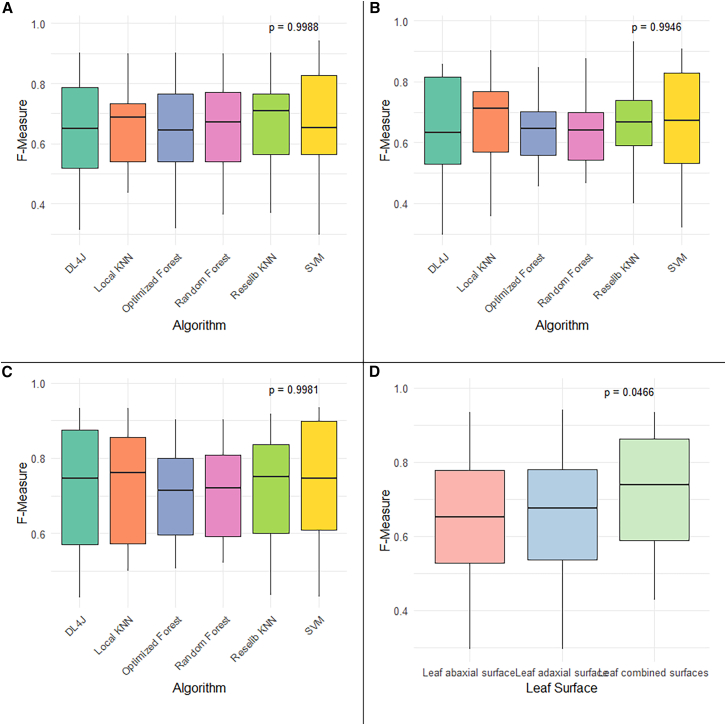
Performance of classification algorithms across different leaf surfaces (A–C) Distribution of *F*-measure values for different classification algorithms applied to identifying the folk types of ayahuasca vine, *B. caapi* and *D. cabrerana*, considering distinct leaf surfaces or combined of them. (D) Anova test.

The analysis of variance based on the *F*-measure indicated no statistically significant differences among the algorithms in the specific evaluations for adaxial, abaxial, and combined (adaxial and abaxial) leaf surfaces (*p* value ≥ 0.05; Figures 2A, 2B, and 2C). Therefore, the algorithm performances in classifying the folk types of *B. caapi* were not attributable to systematic differences among classifiers.

Across the adaxial dataset, mean *F*-measure values ranged from 0.640 to 0.668, with broad overlap of 95% confidence intervals among algorithms (e.g., Rseslib KNN: 0.668, 95% CI 0.556–0.781; SVM: 0.668, 95% CI 0.526–0.809; and DL4J: 0.649, 95% CI 0.514–0.784). The magnitude of the effect of algorithm choice was negligible (η^2^ = 0.004; ω^2^ ≈ 0). For the abaxial dataset, mean *F*-measure values varied between 0.631 and 0.672 (local KNN: 0.672, 95% CI 0.552–0.792; Rseslib KNN: 0.665, 95% CI 0.546–0.784; and SVM: 0.654, 95% CI 0.505–0.803), again showing substantial overlap of confidence intervals and a negligible algorithm effect (η^2^ = 0.008; ω^2^ ≈ 0). For the combined-surface dataset, overall performance increased (means 0.701–0.730), but algorithm-related differences remained negligible (η^2^ = 0.005; ω^2^ ≈ 0), with the highest mean values observed for SVM (0.730, 95% CI 0.600–0.859) and local KNN (0.727, 95% CI 0.612–0.842).

A significant effect of leaf surface (adaxial, abaxial, and combined) on classification performance was detected based on mean *F*-measure values, as indicated by the ANOVA test (*F* = 3.119; *p* value = 0.0466; Figure 2D). Although Tukey’s post hoc test did not identify statistically significant pairwise differences (e.g., combined vs. adaxial: difference = 0.0027; *p* value = 0.9953), the comparisons between combined and abaxial surfaces (difference = 0.0650; *p* value = 0.0726) and between combined and adaxial surfaces (difference = 0.0623; *p* value = 0.0895) approached the 5% significance threshold. These findings highlight the potential benefit of incorporating both adaxial and abaxial leaf surface information to improve classification accuracy of *B. caapi* folk types.

Even without significant statistical differences (*p* value of adaxial = 0.999, abaxial = 0.995, and combined = 0.998) among the six algorithms, the accuracies among them were medium to high (from 60% to 70%) and increased consistently in the combined leaf surfaces analysis (from 64% to 70%) related to adaxial (from 61% to 64%) and abaxial (from 62% to 65%) surface accuracies (Table 1). These accuracy increases showed the potential integration of both leaf surfaces, which is looking to improve the model’s performance. The SVM algorithm stood out with the highest accuracy (70%) and the deep learning 4J had the lowest values (64%).

**Table 1:** Accuracies of correct classification were not statistically significant (*p* value ≥ 0.05) of folk types related to *B*. *caapi* and *D*. *cabrerana* based on leaf surface using six algorithms from three classifiers

Classifier	Algorithm	Adaxial leaf surface (%)	Abaxial leaf surface (%)	Combined leaf surfaces (%)
Lazy	local KNN	63	65	69
	Rseslib KNN	64	65	68
Trees	optimized forest	61	64	68
	random forest	62	63	68
Function	SVM	63	63	70
	deep learning 4J	60	62	64

KNN, k-nearest neighbor algorithm; SVM, support vector machine.

Even though the analysis of variance did not reveal statistically significant differences between the algorithms (*p* value ≥ 0.05), the SVM algorithm for combined leaf surfaces was adopted for further interpretation in this study because it showed the highest accuracy (70%) for correct classification, the highest mean *F*-measure (0.694), and the strong performance in the area under the ROC curve (0.890) (Data S1). Additionally, SVM provided balanced classification across different folk types, confirming its consistency in representing the data obtained from integrating both leaf surfaces.

### Confusion matrix and classification accuracy by folk type

**Figure 3:**
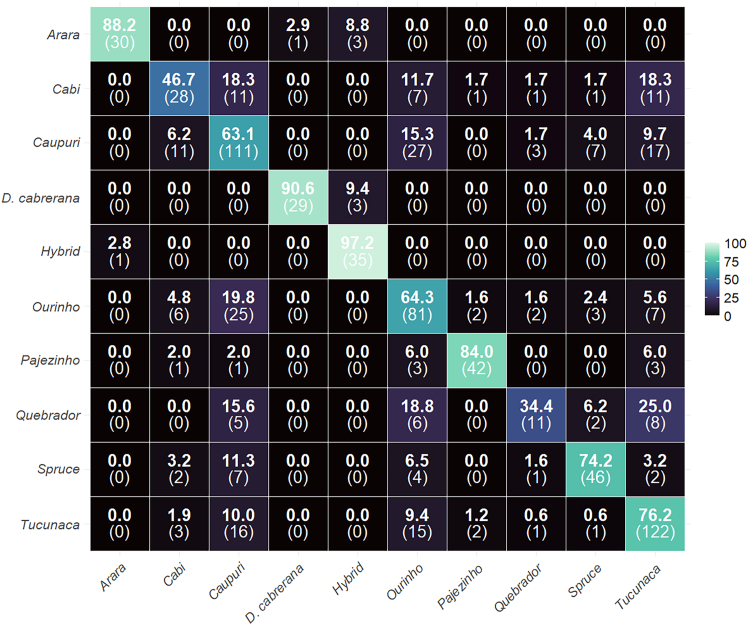
Confusion matrix for the classification of *B. caapi* folk types and *D. cabrerana* Percentage of accuracy and mismatch among *D. cabrerana* and the folk types of *B. caapi* (Arara, Cabi, Caupuri, Caupuri-de-nós-longos (Spruce), Hybrid, Ourinho, Pajezinho, Quebrador and Tucunaca), obtained from the SVM algorithm based on combined leaf surface pictures. The numbers on the diagonal represent the correct percentage of classifications for each folk type, while the other ones in the same line represent the incorrect percentage of classifications. The numbers inside the parentheses represent absolute leaf numbers for each folk type.

The confusion matrix (Figure 3) using the SVM algorithm and the combined (adaxial and abaxial) leaf images dataset demonstrated the assignment performance in classification (Figure 3). Overall, the algorithm achieved remarkable accuracy in correctly identifying several folk types, with values reaching 97% for specific classes. In contrast, others exhibited greater misclassification (34%).

The folk type *Hybrid* (*n*_P_ = 3, *n*_L_ = 18) had the highest accuracy (97%) with one confusion with the folk type *Arara* (Figure 3). *D. cabrerana* (*n*_P_ = 3, *n*_L_ = 18) had the second highest accuracy (91%) with three confusions with the *Hybrid*. Highlighting that morphological leaf surfaces discriminated it from *B. caapi*, justifying the use of *D. cabrerana* as an external group. *Arara* (*n*_P_ = 1, *n*_L_ = 17) and *Pajezinho* (*n*_P_ = 2, *n*_L_ = 25) also achieved high leaf classification accuracies of 88% and 84%, respectively. The vine folk types *Caupuri-de-nós-longos* (*n*_P_ = 1, *n*_L_ = 31) and *Tucunaca* (*n*_P_ = 11, *n*_L_ = 80) were good for high classification accuracy, respectively, 74% and 76%. *Caupuri* (*n*_P_ = 11, *n*_L_ = 88) and *Ourinho* (*n*_P_ = 9, *n*L = 63) had medium leaf accuracy classification, respectively, 63% and 64%. Meanwhile, the folk types *Quebrador* (*n*_P_ = 3, *n*_L_ = 16%–34%) and *Cabi* (*n*_P_ = 4, *n*_L_ = 30%–47%) had the lowest leaf accuracy classification, respectively, 34% and 47%, with the greatest number of confusions among the other six folk types (Figure 3).

### Similarity network of *Banisteriopsis caapi* folk types

**Figure 4:**
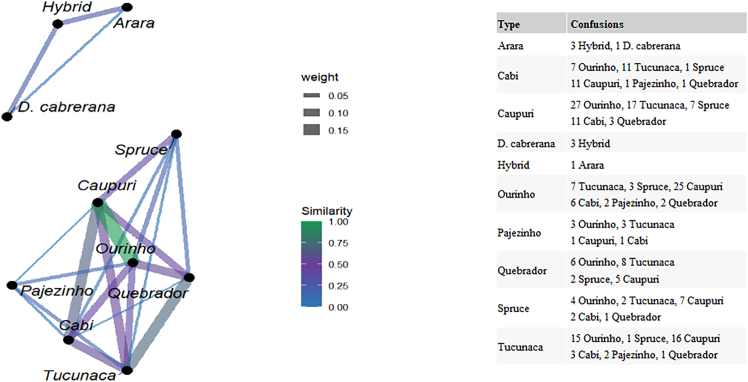
SN of *B. caapi* folk types and *D. cabrerana* The network is based on the SVM algorithm, using the results for combined leaf surfaces.

To better visualize the relationships among the folk types based on SVM model performance, a similarity network (SN; Figure 4) was built which is equivalent to a confusion matrix (Figure 3). In the SN graphic, the nodes represent the folk types and the weighted edges reflect the degree of similarity inferred by the model where the weighted edges highlight the pairwise sample similarities.^69^ The thicker the line, the greater the number of confusions or misclassification patterns among the folk types. This representation reveals notable patterns of similarity contributing to classification of morphological folk types (Figure 4). Although not based on strict statistical distances, the spatial arrangement of nodes offers a geometric approximation of the closeness or separation between clusters. The closer the nodes are, the more similar they are, and the thicker the edge thickness.

Two clusters of folk types were distinct (Figure 4). The first cluster comprised *D. cabrerana*, *Hybrid*, and *Arara* and their respective connections; the second cluster includes the remaining seven folk types (*Cabi*, *Caupuri*, *Caupuri-de-nós-longos*, *Pajezinho*, *Ourinho*, *Quebrador*, and *Tucunaca*) and their connections (Figure 4). Considering *D. cabrerana*, *Hybrid*, and *Arara* as an interconnected cluster, this result also aligned with their high classification accuracy (91%, 97%, and 88%, respectively—Figure 3). Furthermore, the misclassifications observed for *D. cabrerana*, *Hybrid*, and *Arara* (Figure 3) happened only among themselves, with no confusion involving other folk types.

For the other cluster in the SN graphic, the folk types (*Cabi*, *Caupuri*, *Caupuri-de-nós-longos*, *Pajezinho*, *Ourinho*, *Quebrador*, and *Tucunaca*) displayed more confusion revealing a higher level of similarity. The strongest connections, indicated by thicker and shorter lines in greenish tones, were observed between *Ourinho* and *Caupuri* (*n* = 25), *Cabi* and *Caupuri* (*n* = 11), and *Quebrador* and *Tucunaca* (*n* = 8; Figure 4).

The folk types *Ourinho* and *Tucunaca* exhibited the highest and maximum number of confusions within the group (six each; Figure 4), reflecting the highest number of misclassifications (Figure 3). However, these connections are represented by thinner lines and colors closer to blue, indicating weak connections, that is, misclassifications with low percentages (≤10%) (Figures 3 and 4).

*Quebrador* and *Cabi* showed the lowest classification accuracies by the SVM algorithm (37% and 34%, respectively; Figure 3) and displayed multiple connections (from 4 to 6) (Figure 4). For both, the presence of edges with greenish tones and thickness values above 0.10 stands out, reflecting the confusion patterns also observed for these folk types, especially between *Quebrador* and *Tucunaca* (25%), and between *Cabi* and *Caupuri* and *Tucunaca* (18%).

The limited number of samples available for these categories could partially explain these results. Many leaf images were excluded due to poor integrity, as commonly occurs with herbarium specimens. From an initial dataset of 1,646 leaf images, only 768 were retained after quality filtering. This reduction disproportionately affected certain folk types. *Cabi* reduced from 236 to 60 usable images, and *Quebrador* from 122 to 32. In terms of individual plants, the dataset went from 58 to 47 specimens, with *Cabi* being the most affected, dropping from 10 to 4 plants, while other folk types lost were from one to two plants. These imbalances likely limited the model’s ability to generalize morphological patterns for these types, contributing to their lower classification performance.

## Discussion

*B. caapi is* the primary plant used in the ritual preparation of ayahuasca, serving as a foundational subject for studies that bridge scientific inquiry and traditional knowledge. Regarded as sacred by numerous Amazonian peoples, *B. caapi* embodies not only spiritual and symbolic relevance but also considerable ethnobotanical value, having been recognized and managed for generations by traditional communities and religious groups.^2^^,^^4^ The historical record of ayahuasca use extends throughout the northwestern Amazon, encompassing more than 30 ethnic groups across Brazil, Bolivia, Colombia, Ecuador, and Peru, as well as the Orinoco region in Venezuela and the Pacific coast of Colombia and Panama.^7^^,^^8^^,^^9^ However, it is important to note that ayahuasca is not used uniformly across all Indigenous communities, and estimates indicate that only about 10% of global ayahuasca users belong to Indigenous populations.^10^

In the second half of the 20th century, the ritual use of ayahuasca expanded beyond Indigenous contexts and became institutionalized by syncretic Brazilian religions such as União do Vegetal and Santo Daime.^3^^,^^70^ Since then, ayahuasca has diffused across more than 50 countries, where it is now used in religious, therapeutic, and scientific settings.^3^^,^^11^^,^^70^ Recent estimates suggest that over four million people worldwide have consumed ayahuasca, with approximately 820,000 new users and 5.5 million doses recorded in 2019 alone.^71^^,^^72^ This global expansion has raised ethical and ecological concerns regarding the commodification of ayahuasca, overharvesting of its botanical constituents, and marginalization of Indigenous epistemologies, reinforcing the need to preserve both the plant species and the traditional knowledge systems in which they are embedded.^4^^,^^10^^,^^73^

This study lies in its pioneering approach to recovering a folk taxonomy using machine learning, rather than focusing on traditional botanical taxa (e.g., different genera or families). This study contributes to this effort by evaluating whether machine learning algorithms can recover folk classifications of *B. caapi* based on leaf morphology. Recent studies have demonstrated anatomical and genetic differentiation among *B. caapi* ethnotaxa, often preserved in ceremonial contexts through oral transmission and experiential use.^4^^,^^16^ Our findings support the validity of these local classifications and confirm that, despite subtle differences, leaf morphology can distinguish traditional types, including differentiating *B. caapi* from closely related genera such as *Diplopterys*. Thus, this work not only offers a technical contribution to plant taxonomy but also demonstrates how combining morphology, ethnobotanical knowledge, and data science can yield valuable advances in the automated classification of culturally important plants.

Among the six evaluated algorithms, all showed medium to high accuracy (60%–70%), with performance improving when using combined adaxial and abaxial leaf surfaces. While ANOVA revealed no statistically significant differences among the algorithms, the SVM classifier stood out with the highest accuracy (70%), the best *F*-measure, and strong ROC curve performance (Data S1). These findings indicate that leaf morphology contains sufficient diagnostic information to recover most folk classifications, validating the relevance of traditional taxonomies and highlighting machine learning as a promising tool for automated plant identification.

When compared with previous studies on automated plant identification,^25^^,^^26^^,^^29^ the classification accuracies obtained in this study (60%–70%; Table 1) are lower, but remain consistent with the increased difficulty of discriminating closely related taxa and ethnobotanical categories using only leaves. Here, nine folk types belonging to a single species of *B. caapi* were compared using herborized leaves. In contrast, the same machine learning approach applied to 30 tree species from 27 genera and 19 families achieved accuracies above 90% when based on fresh leaves,^25^ indicating that broader taxonomic diversity and stronger morphological contrasts tend to improve classification performance.

In general, studies applying machine learning algorithms such as SVM and random forest frequently report high accuracies (>90%) in interspecific contexts, including seed identification and medicinal plant recognition at the species, genus, or family level.^26^^,^^29^^,^^46^^,^^74^ These approaches typically rely on fresh material or images acquired under controlled conditions, in which morphological differences are more pronounced. By contrast, the present study addresses intra-specific classification of folk types based on herbarium leaf material, a context characterized by subtle, continuous, and highly overlapping morphological variation, and therefore by greater taxonomic, ethnobotanical, and sampling complexity.^75^

The lack of statistically significant differences between the performance of the tested algorithms (as shown by the ANOVA test) suggests that the morphological signal present in B. caapi leaf exsiccates is robust and consistently captured across different computational architectures. This convergence implies that the classification is grounded in stable biological traits rather than being an artifact of a specific mathematical model. For practical applications, this robustness supports the implementation of tools such as the VCode Ayahuasca software for the identification and classification of ayahuasca-related taxa from herbarium leaf images. Furthermore, providing a multi-algorithm approach within the software’s GUI allows researchers to cross-validate results, enhancing the reliability of folk taxonomy recovery in botanical collections.

Previous studies indicate that machine learning algorithms are potentially effective tools for the identification of plant species.^25^^,^^74^^,^^76^^,^^77^^,^^78^ There is not yet a universally superior model; the choice of the most suitable algorithm is directly related to the characteristics of the dataset and the nature of the problem being investigated.^30^

In our study, the success of algorithm models can be attributed to its ability to detect subtle patterns in high-dimensional datasets, a feature particularly suited to classifying leaf images where differences are not always visually obvious.^79^ Combining both leaf surfaces enhanced the representation of morphological diversity; since features like color, shape, and texture vary across leaf surfaces and may provide complementary information.^80^ The bifacial nature of angiosperm leaves adds further value, as the adaxial surface is often more exposed and uniform, while the abaxial surface presents the venation and a high diversity of trichomes and stomata that are taxonomically informative.^81^^,^^82^ Our results confirm that integrating both surfaces increased classification accuracy. In *B. caapi*, the abaxial side is particularly useful due to its clearer venation and gland positions, aiding differentiation.

The confusion matrix (Figure 3) shows high performance for certain types such as *Hybrid* (97%), *D. cabrerana* (91%), and *Arara* (88%), while *Quebrador (*34%) and *Cabi* (47%) exhibited lower accuracy due to greater morphological overlap (Figure 3). These outcomes mirror the challenges of classifying taxa with intraspecific variation, especially when only leaf morphology is considered.

The SN (Figure 4) reinforces the confusion matrix findings, with two distinct clusters: one comprising *D. cabrerana*, *Hybrid*, and *Arara*; and another including *Cabi*, *Caupuri*, *Caupuri*-de-nós-longos, *Pajezinho*, *Ourinho*, *Quebrador*, and *Tucunaca*. These patterns reflect both morphological proximity and misclassification tendencies. Stronger connections between types such as *Caupuri* and *Ourinho*, and *Cabi* and *Caupuri*, point to shared morphological traits, possibly in both leaves and stems.^4^

The Angiosperm Phylogeny Group^83^ emphasize that plant morphology remains a central element for taxonomic distinction and identification, being widely recognized as a foundational basis for species classification. This relevance is reflected in the results of the present study, in which *B. caapi* folk types such as *Hybrid* and *Arara* showed high correct classification rates, exceeding 88% (Figure 3), demonstrating that leaf morphological features provide sufficient information to distinguish these groups. However, species identification is not always straightforward, especially when there is overlap in morphological attributes among taxa.^83^

Previous folk classifications partially align with these clustering. For example, Oliveira et al.^4^ presented a Venn diagram to explain the categorization of vines by folk taxonomists. In this diagram, *Caupuri*, *Caupuri-de-nós-longos*, and *Cabi* are closely related due to their inflated nodes (Figures 1B, 1C, and 1J), while *Arara* appears within the *Tucunaca* group. *Arara* could form a distinct cluster, separate from *Caupuri* and *Tucunaca*, suggesting that the classification based on abaxial leaf surface images by AI does not necessarily follow previously established patterns.^84^ In contrast, the clustering, based on DNA barcoding on *B. caapi* identifies *Arara* as a lineage within *Caupuri*, whereas *Ourinho* is more closely related to *Tucunacas*.^16^

Some groups, such as *Quebrador* (*n*_P_ = 3, *n*_L_ = 16%–34%) and *Cabi* (*n*_P_ = 4, *n*_L_ = 30%–47%), showed low classification performance (Figure 3), even though they were genetically related based on DNA barcode and stem morphology.^16^ This pattern is most plausibly explained by the high degree of morphological overlap between these two folk types and several other categories, which likely limited the algorithms’ ability to extract distinctive leaf-based patterns. Indeed, both *Cabi* and *Quebrador* presented the largest number of confusions with multiple folk types, but no confusion between them indicating intrinsic difficulty in their morphological delimitation based on leaves alone.

Many herbarium leaves were excluded due to poor quality, reducing available samples from 1,646 to 768 images. *Cabi* and *Quebrador* were disproportionately affected (from 236 to 60 and from 122 to 32, respectively), potentially limiting the model’s ability to learn distinct patterns. Although the final sample sizes were not the smallest in the dataset, the substantial reduction in available images may have further constrained model training and amplified the effects of morphological similarity. Therefore, the lower accuracies observed for these two folk types likely reflect a combination of true morphological ambiguity and limitations imposed by the available sampling, suggesting that both larger datasets and additional diagnostic traits will be required to resolve these cases fully.

Traditional *B*. *caapi* classifications emphasize stem morphology (the primary basis for folk classification) and brew characteristics, but leaf traits are increasingly recognized.^4^^,^^16^^,^^84^ *Pajezinho* is associated with high vein density, and *Caupuri-de-nós-longos* with specific gland arrangements. *Arara* is noted for its vibrant leaf coloration, a trait that may explain its high classification accuracy.^4^ However, the model did not reflect expected relationships, such as between *Tucunaca* and *Ourinho*, suggesting that other traits beyond leaf morphology are required for full resolution.

Challenges in automated taxonomy remain, particularly with respect to image quality and intraspecific variation.^85^ The presence of fungal infections, particularly in *Quebrador*, may affect classification, but also provide distinctive features detectable by machine learning. These biological complexities highlight the importance of integrating multiple sources of data to improve accuracy and generalizability.

This study aligns with broader findings that combining morphological traits with machine learning improves taxonomic resolution.^25^^,^^80^ Even though all images were leaf-based, the system achieved notable success in differentiating closely related types within *B. caapi*. In particular, the high accuracy in classifying *D. cabrerana* underscores the potential of machine learning to support genus-level distinctions in morphologically complex groups. Machine learning proved to be an ideal tool for this study because it transcends the limitations of human visual perception in identifying intraspecific morphological overlaps. By processing multidimensional data from leaf exsiccates, the algorithms identified patterns in leaf architecture that persist even after drying, altering leaf color and weakens the leaf’s integrity. This computational approach effectively bridges the gap left by the absence of stem and reproductive traits in herbarium samples, and by the restricted availability of well-documented vouchers for some folk types, providing a standardized, objective method for validating folk taxonomies previously considered inaccessible through leaf morphology alone.

The limitation of using only leaf images highlights the importance of incorporating multiple sources of information to improve the accuracy of differentiation. Studies have shown that integrating various morphological characteristics with computer vision techniques can significantly enhance the discrimination of morphologically similar botanical groups.^86^^,^^87^

Despite some ambiguities, the results support the validity of many folk classifications. Groups like *Arara*, *Hybrid*, and *Caupuri* exhibited high classification rates (Figure 3), even within a single species context, reinforcing the strength of traditional knowledge. Incorporating additional data, such as stem traits, chemical profiles, and genetic markers could enhance model accuracy, especially for less distinct types, and help better understand the biological basis of traditional classifications in ayahuasca and other ethnobotanically important species. Expanding the dataset to include additional specimens and a more balanced representation of all folk types may further improve model robustness and reduce potential classification bias.

Future research should explore interdisciplinary approaches combining machine learning with spectroscopy, anatomy, and molecular data to enhance resolution. Integrating these methodologies can create more robust classification frameworks, contributing not only to scientific knowledge but also to the conservation of biodiversity and cultural heritage. In this broader context, herbarium collections and digital herbaria serve as strategic resources for developing and validating automated plant identification tools applicable to other culturally or taxonomically challenging species.

This study demonstrates that machine learning algorithms, particularly SVM, are effective in distinguishing *B. caapi* folk types based on leaf morphology, even in the presence of high intraspecific variation. The combination of adaxial and abaxial leaf surfaces substantially improved classification accuracy, achieving 70% overall and over 90% for certain folk types such as *Hybrid*, *Arara*, and *D. cabrerana*. However, folk types like *Cabi* and *Quebrador* exhibited lower accuracy due to considerable morphological overlap, suggesting that leaf morphology alone may not be sufficient for all cases.

These findings validate the relevance of traditional folk taxonomies and demonstrate the potential of machine learning, when informed by ethnobotanical knowledge, as a complementary tool for automated plant identification. This approach contributes not only to biodiversity conservation but also to the preservation of cultural knowledge associated with ayahuasca.

Despite the promising outcomes, future studies should consider incorporating additional morphological traits, expanding the dataset to include more individuals and broader geographic variation. Such efforts will enhance the robustness, accuracy, and applicability of this methodology. Ultimately, this work paves the way for the integration of artificial intelligence in ethnobotany and taxonomy, offering new tools for scientific discovery and cultural heritage protection.

### Limitations of the study

This study evaluated the ability of machine learning algorithms to recover folk classifications of *Banisteriopsis caapi* based exclusively on morphological traits extracted from herbarium leaf images. Because herbarium specimens typically lack stems and reproductive structures, the analysis relied solely on leaf morphology, which may not capture the full range of diagnostic traits used in traditional and botanical classifications. Additionally, the dataset included an uneven number of specimens among folk types, and several leaf images had to be excluded due to mechanical damage commonly observed in herbarium material, which reduced the sample size for some categories. These limitations may have contributed to lower classification accuracy for certain folk types exhibiting high morphological overlap. Future studies integrating additional traits such as stem morphology, anatomical characters, chemical profiles, and broader geographic sampling may further improve the resolution of automated classification approaches.

## Resource availability

### Lead contact

Requests for further information and resources should be directed to and will be fulfilled by the lead contact, Scheila Cristina Biazatti (scheila.biazatti@uni.br).

### Materials availability

This study did not generate new biological materials or unique reagents.

### Data and code availability

• The dataset used in this study is available from the lead contact upon reasonable request.
• The VCodeAyahuasca software is open-source and available at https://github.com/DeborahBambil/VCodeAyahuasca.
• Any additional information required to reanalyze the data reported in this paper is available from the lead contact upon request.

## Acknowledgments

The authors thank the Federal University of Rondonia for the qualification license granted to Professor Scheila Cristina Biazatti. The authors would also like to thank the Dean of Human Resources at the University of Brasília (DGP/UnB), the Graduate Program in Botany at the University of Brasília (PPGBot/UnB), and the Federal District Research Support Foundation (FAP-DF) for their support of our research. S.C.B. received qualification license from Universidade Federal de Rondônia (Portaria N° 640/2022/GR/UNIR, de 09 de setembro de 2022). 10.13039/501100003593Conselho Nacional de Desenvolvimento Científico e Tecnológico (CNPq) provided the Research Productivity scholarships to R.C.O. (Proc. 302213/2019-8) and Fundação de Pesquisa do Distrito Federal (10.13039/501100005668FAPDF) also provide financial assistance (grant numbers 0193000881-2015, 0193001773-2017, and 00193-00002220/2023-16). The authors gratefully acknowledge the financial support provided by the DPI/BCE/UnB under Public Notice No. 001/2026 DPI/BCE/UnB.

## Author contributions

Conceptualization, S.C.B. and R.C.O.; methodology, S.C.B., D.B., R.M., L.F.A.F., and R.C.O.; formal analysis, S.C.B., D.B., R.M., L.F.A.F., and R.C.O.; investigation, S.C.B. and R.C.O.; data curation, S.C.B., D.B., and R.C.O.; writing – original draft preparation, S.C.B. and R.C.O.; writing – review and editing, S.C.B., D.B., R.M., L.F.A.F., and R.C.O.; visualization, S.C.B., D.B., R.M., L.F.A.F., and R.C.O.; supervision, L.F.AF. and R.C.O.; project administration, S.C.B. and R.C.O. All authors have read and agreed to the published version of the manuscript.

## Declaration of interests

The authors declare no competing interests.

## Declaration of generative AI and AI-assisted technologies in the writing process

During the preparation of this work, the authors used ChatGPT, DeepL, and Grammarly to assist in improving the scientific writing, translation accuracy, and grammar of the manuscript. After using these tools, the authors reviewed and edited the content as needed and take full responsibility for the content of the published article.

## STAR★Methods

### Key resources table

**Table:** 

REAGENT or RESOURCE	SOURCE	IDENTIFIER
**Biological samples**		

Herbarium specimens of *Banisteriopsis caapi*	UB Herbarium, University of Brasília	UB Herbarium Collection
Herbarium specimens of *Diplopterys cabrerana*	UB Herbarium, University of Brasília	UB Herbarium Collection

**Software and algorithms**		

VCodeAyahuasca software	GitHub	https://github.com/DeborahBambil/VCodeAyahuasca
Weka data mining software	University of Waikato	https://www.cs.waikato.ac.nz/ml/weka
R statistical environment	R Core Team	https://www.r-project.org

**Other**		

iPhone 12 mini (camera device)	Apple Inc.	https://www.apple.com

### Experimental model and study participant details

Dried and herborized mature leaves (*n*_L_ = 384) from 47 vine plants (Malpighiaceae) in the herbarium UB at the University of Brasília were photographed (iPhone 12 mini) on integral or partially abaxial and adaxial surfaces (Table S1). The specimens are preserved in accordance with the standard curatorial guidelines of the UB Herbarium, ensuring their long-term conservation and integrity. Access to the material was granted through formal institutional authorization, supported by a signed Informed Consent Form (ICF; Portuguese: Termo de Consentimento Livre e Esclarecido – TCLE), in accordance with Brazilian ethical and legal frameworks regulating access to biological collections and associated traditional knowledge. Over 95% of these vine plants (*n*_P_ = 45, *n*_L_ = 384) were folk types of *B. caapi*: *Arara* (*n*_P_ = 1, *n*_L_ = 17), *Cabi* (*n*_P_ = 4, *n*_L_ = 30), *Caupuri* (*n*_P_ = 11, *n*_L_ = 88), *Caupuri-de-nós-longos* (*Spruce* - *n*_P_ = 1, *n*_L_ = 31), *Ourinho* (*n*_P_ = 9, *n*_L_ = 63), *Pajezinho* (*n*_P_ = 2, *n*_L_ = 25), *Quebrador* (*n*_P_ = 3, *n*_L_ = 16), *Tucunaca* (*n*_P_ = 11, *n*_L_ = 80), and a supposed *Hybrid* (*n*_P_ = 3, *n*_L_ = 18). The last two vines, from *Diplopterys cabrerana* (Cuatrec.) B.Gates, were used as an external group (*n*_p_ = 2, n_L_ = 16), because it is phylogenetically close to *B. caapi*,^18^^,^^41^^,^^42^ and is also used in the preparation of ayahuasca as a substitute for *P. viridis*.^4^^,^^43^ Given the nature of the study using herborized leaf specimens from monoecious/hermaphroditic vine species, the influence of sex or gender is not applicable to our results.

The folk types analyzed in this study were selected because they correspond to the set of ethnotaxa consistently represented in the UB herbarium collection assembled by Oliveira et al.,^4^ for which sufficient, well-preserved leaf material was available for morphometric analyses. Thus, the selection reflects the diversity of folk categories effectively documented and herborized, rather than *a priori* choice of taxa.

All these leaf donor plants were collected in seven states in Brazil (AC, DF, GO, MG, PA, RJ, and TO). The older exsiccates date from 2017, and the others range from 2019 to 2022. Most of these vine plants (*n* = 43) were cultivated, and four were native (Table S1).

The folk types were classified into two clusters based on whether the stem had inflated nodes. *Caupuri* (*Caupuri-de-nós-longos*, *Cabi*) with stems with inflated nodes and teas with greater physical effect, and the second cluster represented by *Tucunaca* with stems without inflated nodes with weaker teas.^4^ The same clusters happened on stem anatomy of those folks.^4^

### Method details

Initially, the dataset comprised more than 3000 images of adaxial and abaxial leaf surfaces representing nine folk types of *B. caapi* and one related taxon used as an outgroup. Preliminary analyses of the full dataset yielded low classification accuracy (∼50%), prompting quality-based filtering of the images. In the first filtering step, images exhibiting severe mechanical damage, extensive loss of leaf tissue, folds, or poor image quality were removed, leaving 1646 images representing the ten taxa. Although this initial filtering improved model performance, accuracy remained suboptimal (∼60%). Therefore, a second and more stringent filtering step was applied, in which only intact leaves were retained, allowing at most minor apical loss of the leaf blade. This second filtering reduced the dataset to 768 images, with most taxa losing approximately 50% of their images (up to 74% in *Cabi* and 73% in *Quebrador*, and as little as 15% in *Arara* and *D. cabrerana*). Despite this substantial reduction in sample size, all taxa and all classification algorithms showed consistent improvements in model fit and classification accuracy in the final dataset.

The leaves were photographed on both surfaces (adaxial and abaxial), and the leaf sample numbers ranged from 16 to 88 leaves for each taxon. Subsequently, color, shape, texture and filters features were extracted using the VCode Ayahuasca (Available at https://github.com/DeborahBambil/VCodeAyahuasca). The VCodeAyahuasca software features an intuitive graphical user interface (GUI) that streamlines the analysis process. To ensure full reproducibility, all feature extraction procedures and image filters are executed automatically using the software’s built-in default parameters and thresholds, requiring no manual configuration from the user. The extracted features include primary colors (RGB): red, green, and blue and HSV (hue, saturation, value), representing perceptible color, saturation intensity, and brightness.

The CIELab color model, developed by the International Commission on Illumination (CIE), uniformly represents colors for human perception.^44^ L∗ (Lightness) indicates luminosity (0–100), a∗ represents the green-red axis, and b∗ represents the blue-yellow axis. Hu moments comprise seven invariant moments for shape analysis and pattern recognition, invariant to transformations like translation, scaling, and rotation.^45^

Histogram of Oriented Gradients (HOG) extracts image features based on gradient intensity distribution, identifying objects by tracing tonal changes via vectors distributed across image points.^46^ The co-occurrence matrix analyzes image texture by evaluating pixel pair frequencies at specific distances and angles, seeking correlations in surface properties like roughness, smoothness, granularity, luminosity, homogeneity, contrast, and dissimilarity.^47^ Local Binary Pattern (LBP) describes textures by comparing pixel intensity values with neighboring pixels.^48^

Furthermore, additional feature extractors were explored: Gabor Filter, based on harmonic functions modulated by a Gaussian distribution, enabling detection of local frequency and orientation variations.^49^ Canny Edge Detection identifies edges using Gaussian filtering for noise reduction, gradient intensity calculation, non-maximum suppression, and dual thresholding for strong and weak edge identification.^50^

The Fourier Transform is a fundamental mathematical tool for signal analysis and image processing, decomposing time/space-domain signals into frequency components. This technique converts the original function into a spectral representation, facilitating periodic pattern identification and dominant frequency analysis. Fourier transforms enable frequency-domain filtering, compression, and restoration in image processing. The process involves calculating frequency component amplitudes and phases via integral/discrete transforms, followed by inverse Fourier Transform reconstruction. Its efficiency in capturing global and local frequency information makes it essential in scientific applications, such as texture analysis, noise removal, and spectral studies.^51^

The Scharr Filter is an enhanced edge detection technique providing accurate digital image gradient estimation. Derived from the Sobel operator, Scharr utilizes optimized for horizontal and vertical gradient calculation masks, offering sensitivity to subtle intensity changes, particularly in smooth or fine-textured regions. Its mathematical construction reduces quantization-introduced error amplification, ensuring calculated gradient fidelity. This filter excels in applications requiring precise edge detection or detailed structural analysis, such as pattern recognition, object segmentation, and texture analysis.^52^

The Laplacian Filter, widely used in image processing, employs second-order derivatives for edge enhancement and detection. It calculates intensity variation rates in image regions, highlighting abrupt transitions between adjacent pixels. The application involves discrete masks for estimating the intensity of Laplacian function, often preceded by noise-reducing smoothing. This method excels in identifying isotropic edges and operating direction independently, with applications in analyses requiring precise structural feature detection.^53^

The Sobel Filter is a classic edge detection technique based on first-order derivatives. It uses convolutional masks to calculate horizontal and vertical intensity gradients, identifying significant local variations. This approach highlights intensity transitions along edges and contours, emphasizing smooth region variations. Due to its robustness in edge detection, combining sensitivity to intensity transitions with intrinsic noise smoothing, Sobel is widely used in image preprocessing for structural feature analysis, object segmentation, and visual pattern recognition.^54^

The Prewitt Filter is a widely employed edge detection technique utilizing first-order derivatives to estimate intensity gradients. This method applies to discrete convolutional masks oriented along horizontal and vertical axes, identifying significant luminosity transitions. Although like Sobel, Prewitt is characterized by computational simplicity, making it efficient for low-computational-cost applications. It’s beneficial for contour identification in low-noise images, contributing to object segmentation and visual feature extraction in computer vision systems.^55^

### Quantification and statistical analysis

#### Classification algorithms

The Weka data mining tool classified image features using local KNN, rseslib KNN, optimized forest, random forest, DL4j, and SVM algorithms to ensure robust classification and mitigate the risk of overfitting. All models were evaluated using 10-fold cross-validation. In this procedure, the dataset was partitioned into 10 equal subsets; in each iteration, 9 subsets were used for training while the remaining subset served as the validation set. This process was repeated 10 times, ensuring that each leaf sample was used for both training and testing.^56^ Finally, it combines the random forest predictions with those of a cost-sensitive model to generate the final output.^57^

Local KNN (k-Nearest Neighbors) is a variation of the classic k-Nearest Neighbors (KNN) algorithm that emphasizes adapting the model to different regions of the feature space. Instead of using a fixed k value for the entire dataset, local KNN dynamically adjusts k based on the data density.^58^ Rseslib KNN refers to implementing the k-Nearest Neighbors (KNN) algorithm within rseslib, a Java-based library for rough set theory and machine learning. This library provides various tools for data analysis, classification, and feature selection, including an optimized KNN algorithm.

SVM was chosen for its efficiency in high-dimensional margin separation, crucial for distinguishing overlapping folk types. Random Forest and its optimized versions were employed due to their robustness against missing data and noise, a common issue in damaged herbarium specimens. KNN algorithms provided a similarity-based approach akin to traditional botanical comparisons, while DL4J enabled the extraction of complex, non-linear structural features. This multi-algorithmic framework ensures that the recovery of folk taxonomy is not dependent on a single mathematical model, but instead on a consistent biological signal detected across different logical architectures.

Optimized forest is a machine learning algorithm that enhances predictive accuracy by integrating feature selection and ensemble learning. It first identifies a subset of the most relevant features, reducing dimensionality and mitigating overfitting. A random forest is trained using only these selected features, improving the model’s efficiency. Finally, the predictions from the random forest were combined with those from an optimized model that may incorporate cost-sensitive learning, weighted classification, or other ensemble techniques. This approach improves performance, reduces computational complexity, and enhances interpretability, making it particularly useful for classification and regression tasks with high-dimensional data.^59^

DL4j is a multi-layer artificial neural network algorithm for modeling complex problems for image classification and recognition.^60^^,^^61^ The SVM algorithm resolves classification problems by region; a hyperplane separates classes. The hyperplane distance is defined by support vectors establishing a margin within the dataset.^62^

#### Performance evaluation

To assess classification accuracy, ANOVA and Tukey tests will be performed using R software.^63^ Based on the F-measure metric, this approach will compare classification results pairwise, considering harmonic means of true positives, false positives, and false negatives. This analysis was used as a preliminary step to select the algorithm with the best statistical performance and, consequently, the one that best represents the data. For details on the individual performance of the algorithms (Data S1).

In addition to *p*-values, we quantified the magnitude of algorithm-related effects using eta-squared (η^2^) and omega-squared (ω^2^) as effect size measures derived from the one-way ANOVA. To provide precise estimates of model performance, we also computed t-based 95% confidence intervals for the mean F-measure for each algorithm across dataset (adaxial, abaxial, and combined leaf surfaces).

The confusion matrix was used to evaluate the correct classification of *B. caapi* folk types and *D. cabrerana* (Cuatrec.) B.Gates, using abaxial and adaxial leaf surfaces. The matrix was normalized to percentage values and visualized as a heatmap in the R environment using the ggplot2^64^ and viridis^65^ packages. In the latter package, the color intensity reflects the frequency of the classifications. This approach allowed us to identify the folk types with greater precision and those with more significant overlap in classification.

A network graph based on a confusion matrix was built to visualize the similarity relations and confusions among the folk types generated by the best SVM algorithm using the combined (adaxial and abaxial) leaf surfaces. The nodes represent the folk types, and the edges indicate the levels of connection, weighted by the frequency of erroneous classifications. The connections (edges) indicate the levels of similarity, whose thickness varies proportionally to the weight attributed by the correct classifications. The spatial arrangement of the nodes allows approximations and distances between the clusters to be visualized, although not corresponding to an exact metric scale. The analysis was performed in the R environment, using the igraph^66^^,^^67^ and ggraph^68^ packages, with the Fruchterman-Reingold layout to distribute the nodes according to their connections. The edges were colored and adjusted in thickness to reflect the intensity of the connection between the classes. This approach allowed the identification of clustering patterns and morphological similarity, highlighting which folk types are more differentiable and which present higher overlap in the classification.

## Supplemental information

Document S1. Data S1

Table S1. Collection metadata for folk types of *Banisteriopsis caapi* and *Diplopterys cabrerana*
