# Supplementary material for: Machine learning recovers folk classification of Banisteriopsis caapi from herbarium leaves an ayahuasca liana
Source: iScience. 2026 Apr 15;29(5):115753. doi: 10.1016/j.isci.2026.115753 (PMC13145879; doi:10.1016/j.isci.2026.115753)
Supplement: Document S1. Data S1 [file mmc1.pdf]

iScience, Volume *29*

Supplemental information

Machine learning recovers folk classification of *Banisteriopsis caapi* from herbarium leaves an ayahuasca liana

Scheila Cristina Biazatti, Deborah Bambil, Rômulo Môra, Lúcio Flávio de Alencar Figueiredo, and Regina Célia de Oliveira

Data S1. Individual performance of the algorithms and F-measure metric.

LEAF ADAXIAL SURFACE

RESELIB KNN

=== Summary ===

Correctly Classified Instances	247	64.3229 %
Incorrectly Classified Instances	137	35.6771 %
Kappa statistic	0.5806	
Mean absolute error	0.0943	
Root mean squared error	0.2347	
Relative absolute error	55.1838 %	
Root relative squared error	80.3586 %	
Total Number of Instances	384	

=== Detailed Accuracy By Class ===

	TP Rate	FP Rate	Precision	Recall	F-Measure	MCC	ROC Area	PRC Area	Class
	0,840	0,031	0,656	0,840	0,737	0,722	0,968	0,855	pajezinho
	0,663	0,079	0,688	0,663	0,675	0,592	0,851	0,690	tucunaca
	0,476	0,062	0,600	0,476	0,531	0,455	0,794	0,486	ourinho
	0,774	0,020	0,774	0,774	0,774	0,754	0,912	0,620	spruce
	0,567	0,040	0,548	0,567	0,557	0,519	0,838	0,431	cabi
	0,875	0,003	0,933	0,875	0,903	0,900	0,936	0,880	cabrerana
	0,636	0,155	0,549	0,636	0,589	0,458	0,795	0,506	caupuri
	0,313	0,016	0,455	0,313	0,370	0,355	0,803	0,375	quebrador
	0,824	0,011	0,778	0,824	0,800	0,791	0,964	0,775	arara
	0,722	0,011	0,765	0,722	0,743	0,731	0,963	0,700	hibrido
Weighted Avg.	0,643	0,071	0,644	0,643	0,640	0,573	0,852	0,598	

=== Confusion Matrix ===

a	b	c	d	e	f	g	h	i	j	<-- classified as
21	1	1	0	0	0	2	0	0	0	a = pajezinho
3	53	7	0	2	0	14	1	0	0	b = tucunaca
4	6	30	3	2	0	17	1	0	0	c = ourinho
0	0	1	24	4	0	2	0	0	0	d = spruce
1	4	1	1	17	0	6	0	0	0	e = cabi
0	0	0	0	0	14	0	0	1	1	f = cabrerana
2	11	8	2	5	0	56	4	0	0	g = caupuri
1	2	2	1	1	0	4	5	0	0	h = quebrador
0	0	0	0	0	0	0	0	14	3	i = arara
0	0	0	0	0	1	1	0	3	13	j = hibrido

LOCAL KNN

=== Summary ===

Correctly Classified Instances	241	62.7604 %
Incorrectly Classified Instances	143	37.2396 %
Kappa statistic	0.5597	
Mean absolute error	0.0978	
Root mean squared error	0.2301	
Relative absolute error	57.2612 %	
Root relative squared error	78.7909 %	
Total Number of Instances	384	

=== Detailed Accuracy By Class ===

TP Rate	FP Rate	Precision	Recall	F-Measure	MCC	ROC Area	PRC Area	Class
0,840	0,031	0,656	0,840	0,737	0,722	0,951	0,859	pajezinho
0,713	0,095	0,663	0,713	0,687	0,601	0,876	0,739	tucunaca
0,444	0,044	0,667	0,444	0,533	0,476	0,794	0,565	ourinho
0,710	0,023	0,733	0,710	0,721	0,697	0,922	0,653	spruce

	0,433	0,045	0,448	0,433	0,441	0,394	0,851	0,392	cabi
	0,875	0,003	0,933	0,875	0,903	0,900	0,967	0,918	cabrerana
	0,636	0,182	0,509	0,636	0,566	0,422	0,803	0,556	caupuri
	0,313	0,005	0,714	0,313	0,435	0,459	0,803	0,460	quebrador
	0,765	0,008	0,813	0,765	0,788	0,779	0,965	0,877	arara
	0,667	0,014	0,706	0,667	0,686	0,671	0,988	0,732	hibrido
Weighted Avg.	0,628	0,078	0,639	0,628	0,624	0,557	0,862	0,644	

=== Confusion Matrix ===

```
  a  b  c  d  e  f  g  h  i  j   <-- classified as
 21  1  1  0  1  0  1  0  0  0 |  a = pajezinho
  2 57  1  0  1  0 18  1  0  0 |  b = tucunaca
  4  9 28  3  3  0 16  0  0  0 |  c = ourinho
  0  2  0 22  4  0  3  0  0  0 |  d = spruce
  2  2  2  1 13  0 10  0  0  0 |  e = cabi
  0  0  0  0  0 14  0  0  1  1 |  f = cabrerana
  2 11  8  3  7  0 56  1  0  0 |  g = caupuri
  1  3  2  1  0  0  4  5  0  0 |  h = quebrador
  0  0  0  0  0  0  0  0 13  4 |  i = arara
  0  1  0  0  0  1  2  0  2 12 |  j = hibrido
```

OPTIMEZED FOREST

=== Summary ===

Correctly Classified Instances 236 61.4583 %

Incorrectly Classified Instances 148 38.5417 %

Kappa statistic 0.541

Mean absolute error 0.1216

Root mean squared error 0.2339

Relative absolute error 71.208 %

Root relative squared error 80.0769 %

Total Number of Instances 384

=== Detailed Accuracy By Class ===

	TP Rate	FP Rate	Precision	Recall	F-Measure	MCC	ROC Area	PRC Area	Class
	0,800	0,019	0,741	0,800	0,769	0,753	0,980	0,873	pajezinho
	0,725	0,115	0,624	0,725	0,671	0,578	0,878	0,770	tucunaca
	0,492	0,065	0,596	0,492	0,539	0,462	0,837	0,586	ourinho
	0,581	0,025	0,667	0,581	0,621	0,592	0,919	0,709	spruce
	0,233	0,020	0,500	0,233	0,318	0,306	0,896	0,471	cabi
	0,875	0,003	0,933	0,875	0,903	0,900	0,985	0,915	cabrerana
	0,625	0,199	0,482	0,625	0,545	0,392	0,827	0,546	caupuri
	0,313	0,003	0,833	0,313	0,455	0,499	0,865	0,487	quebrador
	0,824	0,008	0,824	0,824	0,824	0,815	0,990	0,895	arara
	0,778	0,014	0,737	0,778	0,757	0,745	0,987	0,652	hibrido
Weighted Avg.	0,615	0,086	0,624	0,615	0,606	0,536	0,885	0,661	

=== Confusion Matrix ===

a	b	c	d	e	f	g	h	i	j	<-- classified as
20	1	2	0	0	0	2	0	0	0	a = pajezinho
0	58	4	1	0	0	16	0	0	1	b = tucunaca
3	8	31	4	2	0	15	0	0	0	c = ourinho
0	2	0	18	2	0	8	1	0	0	d = spruce
2	5	0	1	7	0	15	0	0	0	e = cabi
0	0	0	0	0	14	0	0	1	1	f = cabrerana
1	13	14	3	2	0	55	0	0	0	g = caupuri
1	5	1	0	1	0	3	5	0	0	h = quebrador
0	0	0	0	0	0	0	0	14	3	i = arara
0	1	0	0	0	1	0	0	2	14	j = hibrido

RANDOM FOREST

=== Summary ===

Correctly Classified Instances	240	62.5 %
Incorrectly Classified Instances	144	37.5 %
Kappa statistic	0.5527	
Mean absolute error	0.1216	
Root mean squared error	0.2336	
Relative absolute error	71.1902 %	
Root relative squared error	79.9942 %	
Total Number of Instances	384	

=== Detailed Accuracy By Class ===

	TP Rate	FP Rate	Precision	Recall	F-Measure	MCC	ROC Area	PRC Area	Class
	0,800	0,017	0,769	0,800	0,784	0,769	0,977	0,862	pajezinho
	0,725	0,109	0,637	0,725	0,678	0,589	0,881	0,778	tucunaca
	0,492	0,069	0,585	0,492	0,534	0,455	0,838	0,586	ourinho
	0,613	0,020	0,731	0,613	0,667	0,643	0,921	0,711	spruce
	0,267	0,017	0,571	0,267	0,364	0,358	0,897	0,442	cabi
	0,875	0,003	0,933	0,875	0,903	0,900	0,983	0,909	cabrerana
	0,659	0,203	0,492	0,659	0,563	0,416	0,827	0,546	caupuri
	0,313	0,003	0,833	0,313	0,455	0,499	0,861	0,469	quebrador
	0,882	0,014	0,750	0,882	0,811	0,804	0,991	0,892	arara
	0,667	0,008	0,800	0,667	0,727	0,718	0,986	0,640	hibrido
Weighted Avg.	0,625	0,086	0,639	0,625	0,618	0,550	0,885	0,658	

=== Confusion Matrix ===

```
 a  b  c  d  e  f  g  h  i  j   <-- classified as
20  2  2  0  0  0  1  0  0  0 |  a = pajezinho
 0 58  4  1  0  0 17  0  0  0 |  b = tucunaca
```

3 7 31 2 1 0 18 1 0 0 | c = ourinho

0 2 0 19 2 0 8 0 0 0 | d = spruce

2 6 0 1 8 0 13 0 0 0 | e = cabi

0 0 0 0 0 14 0 0 1 1 | f = cabrerana

1 10 14 3 2 0 58 0 0 0 | g = caupuri

0 5 2 0 1 0 3 5 0 0 | h = quebrador

0 0 0 0 0 0 0 0 15 2 | i = arara

0 1 0 0 0 1 0 0 4 12 | j = hibrido

SVM

=== Summary ===

Correctly Classified Instances	241	62.7604 %
Incorrectly Classified Instances	143	37.2396 %
Kappa statistic	0.5632	
Mean absolute error	0.1636	
Root mean squared error	0.2785	
Relative absolute error	95.7645 %	
Root relative squared error	95.3593 %	
Total Number of Instances	384	

=== Detailed Accuracy By Class ===

TP Rate	FP Rate	Precision	Recall	F-Measure	MCC	ROC Area	PRC Area	Class
0,840	0,022	0,724	0,840	0,778	0,763	0,980	0,755	pajezinho
0,600	0,112	0,585	0,600	0,593	0,484	0,840	0,519	tucunaca
0,571	0,084	0,571	0,571	0,571	0,487	0,853	0,476	ourinho
0,710	0,025	0,710	0,710	0,710	0,684	0,875	0,610	spruce
0,500	0,040	0,517	0,500	0,508	0,468	0,795	0,368	cabi
0,875	0,005	0,875	0,875	0,875	0,870	0,989	0,853	cabrerana

	0,557	0,125	0,570	0,557	0,563	0,435	0,795	0,469	caupuri
	0,250	0,019	0,364	0,250	0,296	0,277	0,787	0,208	quebrador
	0,941	0,003	0,941	0,941	0,941	0,938	0,993	0,904	arara
	0,889	0,011	0,800	0,889	0,842	0,835	0,992	0,796	hibrido
Weighted Avg.	0,628	0,074	0,622	0,628	0,624	0,550	0,858	0,542	

=== Confusion Matrix ===

a	b	c	d	e	f	g	h	i	j	<-- classified as
21	2	1	0	1	0	0	0	0	0	a = pajezinho
4	48	6	1	3	0	15	3	0	0	b = tucunaca
3	5	36	2	3	0	13	0	0	1	c = ourinho
0	2	0	22	2	0	4	1	0	0	d = spruce
1	5	3	1	15	0	3	2	0	0	e = cabi
0	0	0	0	0	14	0	0	0	2	f = cabrerana
0	16	13	5	4	0	49	1	0	0	g = caupuri
0	4	4	0	1	1	2	4	0	0	h = quebrador
0	0	0	0	0	0	0	0	16	1	i = arara
0	0	0	0	0	1	0	0	1	16	j = hibrido

DL4j

=== Summary ===

Correctly Classified Instances	231	60.1563 %
Incorrectly Classified Instances	153	39.8438 %
Kappa statistic	0.5335	
Mean absolute error	0.1001	
Root mean squared error	0.2348	
Relative absolute error	58.6105 %	
Root relative squared error	80.4023 %	
Total Number of Instances	384	

=== Detailed Accuracy By Class ===

	TP Rate	FP Rate	Precision	Recall	F-Measure	MCC	ROC Area	PRC Area	Class
	0,760	0,033	0,613	0,760	0,679	0,658	0,961	0,806	pajezinho
	0,613	0,095	0,628	0,613	0,620	0,522	0,870	0,676	tucunaca
	0,508	0,072	0,582	0,508	0,542	0,461	0,827	0,554	ourinho
	0,677	0,025	0,700	0,677	0,689	0,662	0,907	0,703	spruce
	0,467	0,048	0,452	0,467	0,459	0,412	0,860	0,402	cabi
	0,813	0,003	0,929	0,813	0,867	0,863	0,993	0,927	cabrerana
	0,500	0,159	0,484	0,500	0,492	0,337	0,793	0,496	caupuri
	0,500	0,014	0,615	0,500	0,552	0,537	0,918	0,506	quebrador
	0,824	0,005	0,875	0,824	0,848	0,842	0,991	0,894	arara
	0,944	0,022	0,680	0,944	0,791	0,790	0,990	0,794	hibrido
Weighted Avg.	0,602	0,078	0,604	0,602	0,600	0,523	0,872	0,622	

=== Confusion Matrix ===

a	b	c	d	e	f	g	h	i	j	<-- classified as
19	3	1	0	0	0	1	1	0	0	a = pajezinho
6	49	5	1	2	0	14	1	0	2	b = tucunaca
3	4	32	3	3	0	17	1	0	0	c = ourinho
0	0	0	21	3	0	5	2	0	0	d = spruce
1	4	2	1	14	0	8	0	0	0	e = cabi
0	0	0	0	0	13	0	0	0	3	f = cabrerana
1	18	12	4	8	0	44	0	1	0	g = caupuri
1	0	3	0	1	1	2	8	0	0	h = quebrador
0	0	0	0	0	0	0	0	14	3	i = arara
0	0	0	0	0	0	0	0	1	17	j = hibrido

<u>LEAF ABAXIAL SURFACE</u>

LOCAL KNN

=== Summary ===

Correctly Classified Instances	250	65.2742 %
Incorrectly Classified Instances	133	34.7258 %
Kappa statistic	0.5897	
Mean absolute error	0.0835	
Root mean squared error	0.2254	
Relative absolute error	48.8935 %	
Root relative squared error	77.1914 %	
Total Number of Instances	383	

=== Detailed Accuracy By Class ===

	TP Rate	FP Rate	Precision	Recall	F-Measure	MCC	ROC Area	PRC Area	Class
	0,722	0,014	0,722	0,722	0,722	0,709	0,963	0,731	hibrido
	0,720	0,008	0,857	0,720	0,783	0,772	0,912	0,761	pajezinho
	0,675	0,063	0,740	0,675	0,706	0,634	0,873	0,726	tucunaca
	0,875	0,003	0,933	0,875	0,903	0,900	0,936	0,876	cabrerana
	0,433	0,025	0,591	0,433	0,500	0,471	0,858	0,524	cabi
	0,571	0,100	0,529	0,571	0,550	0,457	0,824	0,559	ourinho
	0,733	0,025	0,710	0,733	0,721	0,697	0,913	0,763	spruce
	0,313	0,019	0,417	0,313	0,357	0,337	0,713	0,311	quebrador
	0,882	0,008	0,833	0,882	0,857	0,851	0,967	0,843	arara
	0,682	0,153	0,571	0,682	0,622	0,499	0,853	0,603	caupuri
Weighted Avg.	0,653	0,071	0,658	0,653	0,652	0,587	0,869	0,654	

=== Confusion Matrix ===

a b c d e f g h i j <-- classified as

13 0 1 1 0 0 0 0 3 0 | a = hibrido

0 18 2 0 1 3 0 0 0 1 | b = pajezinho

1 0 54 0 0 10 1 2 0 12 | c = tucunaca

2 0 0 14 0 0 0 0 0 0 | d = cabrerana

0 1 3 0 13 6 1 0 0 6 | e = cabi

0 2 4 0 3 36 1 1 0 16 | f = ourinho

0 0 1 0 1 1 22 0 0 5 | g = spruce

0 0 3 0 0 3 0 5 0 5 | h = quebrador

2 0 0 0 0 0 0 0 15 0 | i = arara

0 0 5 0 4 9 6 4 0 60 | j = caupuri

RESELIB KNN

=== Summary ===

Correctly Classified Instances	250	65.2742 %
Incorrectly Classified Instances	133	34.7258 %
Kappa statistic	0.5875	
Mean absolute error	0.0808	
Root mean squared error	0.2321	
Relative absolute error	47.3126 %	
Root relative squared error	79.497 %	
Total Number of Instances	383	

=== Detailed Accuracy By Class ===

TP Rate	FP Rate	Precision	Recall	F-Measure	MCC	ROC Area	PRC Area	Class
0,722	0,011	0,765	0,722	0,743	0,731	0,938	0,699	hibrido
0,520	0,011	0,765	0,520	0,619	0,610	0,845	0,616	pajezinho
0,738	0,092	0,678	0,738	0,707	0,626	0,881	0,720	tucunaca
0,875	0,000	1,000	0,875	0,933	0,933	0,937	0,880	cabrerana
0,400	0,031	0,522	0,400	0,453	0,417	0,808	0,458	cabi
0,571	0,078	0,590	0,571	0,581	0,500	0,828	0,517	ourinho

	0,700	0,020	0,750	0,700	0,724	0,702	0,898	0,625	spruce
	0,313	0,011	0,556	0,313	0,400	0,398	0,666	0,362	quebrador
	0,941	0,011	0,800	0,941	0,865	0,861	0,966	0,802	arara
	0,693	0,156	0,570	0,693	0,626	0,504	0,817	0,561	caupuri
Weighted Avg.	0,653	0,074	0,656	0,653	0,648	0,584	0,851	0,610	

=== Confusion Matrix ===

```
  a  b  c  d  e  f  g  h  i  j   <-- classified as
 13  0  1  0  0  0  0  0  4  0 |  a = hibrido
  0 13  2  0  1  5  0  0  0  4 |  b = pajezinho
  1  0 59  0  0  4  1  2  0 13 |  c = tucunaca
  2  0  0 14  0  0  0  0  0  0 |  d = cabrerana
  0  2  4  0 12  4  2  0  0  6 |  e = cabi
  0  0  6  0  2 36  1  2  0 16 |  f = ourinho
  0  0  3  0  3  0 21  0  0  3 |  g = spruce
  0  0  3  0  0  4  0  5  0  4 |  h = quebrador
  1  0  0  0  0  0  0  0 16  0 |  i = arara
  0  2  9  0  5  8  3  0  0 61 |  j = caupuri
```

OPTIMEZED FOREST

=== Summary ===

Correctly Classified Instances	245	63.9687 %
Incorrectly Classified Instances	138	36.0313 %
Kappa statistic	0.5688	
Mean absolute error	0.1192	
Root mean squared error	0.23	
Relative absolute error	69.8089 %	
Root relative squared error	78.7837 %	
Total Number of Instances	383	

=== Detailed Accuracy By Class ===

	TP Rate	FP Rate	Precision	Recall	F-Measure	MCC	ROC Area	PRC Area	Class
	0,667	0,011	0,750	0,667	0,706	0,694	0,989	0,723	hibrido
	0,560	0,011	0,778	0,560	0,651	0,641	0,980	0,827	pajezinho
	0,738	0,106	0,648	0,738	0,690	0,604	0,905	0,785	tucunaca
	0,875	0,008	0,824	0,875	0,848	0,842	0,982	0,915	cabrerana
	0,333	0,011	0,714	0,333	0,455	0,461	0,890	0,506	cabi
	0,508	0,078	0,561	0,508	0,533	0,448	0,847	0,607	ourinho
	0,600	0,023	0,692	0,600	0,643	0,617	0,940	0,740	spruce
	0,313	0,000	1,000	0,313	0,476	0,551	0,802	0,472	quebrador
	0,824	0,011	0,778	0,824	0,800	0,791	0,957	0,886	arara
	0,761	0,183	0,554	0,761	0,641	0,523	0,864	0,627	caupuri
Weighted Avg.	0,640	0,082	0,662	0,640	0,632	0,572	0,898	0,691	

=== Confusion Matrix ===

a	b	c	d	e	f	g	h	i	j	<-- classified as
12	0	0	2	0	0	0	0	4	0	a = hibrido
0	14	4	0	1	4	0	0	0	2	b = pajezinho
0	1	59	0	1	6	1	0	0	12	c = tucunaca
2	0	0	14	0	0	0	0	0	0	d = cabrerana
0	1	5	0	10	2	1	0	0	11	e = cabi
0	1	9	0	1	32	3	0	0	17	f = ourinho
0	0	3	0	0	3	18	0	0	6	g = spruce
0	0	3	0	0	2	0	5	0	6	h = quebrador
2	0	0	1	0	0	0	0	14	0	i = arara
0	1	8	0	1	8	3	0	0	67	j = caupuri

RANDOM FOREST

=== Summary ===

Correctly Classified Instances 242 63.1854 %

Incorrectly Classified Instances 141 36.8146 %

Kappa statistic 0.5595

Mean absolute error 0.1192

Root mean squared error 0.2298

Relative absolute error 69.8299 %

Root relative squared error 78.6997 %

Total Number of Instances 383

=== Detailed Accuracy By Class ===

	TP Rate	FP Rate	Precision	Recall	F-Measure	MCC	ROC Area	PRC Area	Class
	0,667	0,011	0,750	0,667	0,706	0,694	0,988	0,714	hibrido
	0,560	0,011	0,778	0,560	0,651	0,641	0,980	0,825	pajezinho
	0,725	0,106	0,644	0,725	0,682	0,594	0,905	0,783	tucunaca
	0,875	0,005	0,875	0,875	0,875	0,870	0,983	0,912	cabrerana
	0,333	0,008	0,769	0,333	0,465	0,482	0,899	0,539	cabi
	0,492	0,084	0,534	0,492	0,512	0,422	0,851	0,608	ourinho
	0,600	0,025	0,667	0,600	0,632	0,603	0,939	0,741	spruce
	0,313	0,000	1,000	0,313	0,476	0,551	0,787	0,463	quebrador
	0,824	0,014	0,737	0,824	0,778	0,768	0,956	0,896	arara
	0,750	0,186	0,545	0,750	0,632	0,510	0,866	0,644	caupuri
Weighted Avg.	0,632	0,084	0,657	0,632	0,624	0,563	0,899	0,696	

=== Confusion Matrix ===

a b c d e f g h i j <-- classified as

12 0 0 1 0 0 0 0 5 0 | a = hibrido

0 14 4 0 0 4 0 0 0 3 | b = pajezinho

0 1 58 0 1 8 1 0 0 11 | c = tucunaca

2 0 0 14 0 0 0 0 0 0 | d = cabrerana

0	1	5	0	10	2	1	0	0	11	|	e = cabi
0	1	8	0	1	31	4	0	0	18	|	f = ourinho
0	0	3	0	0	3	18	0	0	6	|	g = spruce
0	0	3	0	0	2	0	5	0	6	|	h = quebrador
2	0	0	1	0	0	0	0	14	0	|	i = arara
0	1	9	0	1	8	3	0	0	66	|	j = caupuri

SVM

=== Summary ===

Correctly Classified Instances	241	62.9243 %
Incorrectly Classified Instances	142	37.0757 %
Kappa statistic	0.5616	
Mean absolute error	0.1635	
Root mean squared error	0.2784	
Relative absolute error	95.7746 %	
Root relative squared error	95.3525 %	
Total Number of Instances	383	

=== Detailed Accuracy By Class ===

TP Rate	FP Rate	Precision	Recall	F-Measure	MCC	ROC Area	PRC Area	Class
0,944	0,011	0,810	0,944	0,872	0,868	0,993	0,790	hibrido
0,640	0,020	0,696	0,640	0,667	0,645	0,972	0,665	pajezinho
0,725	0,089	0,682	0,725	0,703	0,622	0,860	0,606	tucunaca
0,875	0,003	0,933	0,875	0,903	0,900	0,983	0,903	cabrerana
0,367	0,048	0,393	0,367	0,379	0,329	0,850	0,283	cabi
0,524	0,103	0,500	0,524	0,512	0,413	0,799	0,404	ourinho
0,600	0,014	0,783	0,600	0,679	0,663	0,909	0,597	spruce
0,250	0,014	0,444	0,250	0,320	0,312	0,730	0,178	quebrador
0,882	0,003	0,938	0,882	0,909	0,905	0,949	0,855	arara

0,625 0,142 0,567 0,625 0,595 0,467 0,837 0,508 caupuri

Weighted Avg. 0,629 0,076 0,630 0,629 0,626 0,556 0,865 0,542

=== Confusion Matrix ===

```
  a  b  c  d  e  f  g  h  i  j   <-- classified as
 17  0  0  0  0  0  0  0  1  0 |  a = hibrido
  0 16  2  0  0  5  0  1  0  1 |  b = pajezinho
  0  1 58  0  2  6  0  1  0 12 |  c = tucunaca
  2  0  0 14  0  0  0  0  0  0 |  d = cabrerana
  0  2  6  0 11  2  1  1  0  7 |  e = cabi
  0  2  7  0  5 33  1  1  0 14 |  f = ourinho
  0  0  2  0  3  2 18  0  0  5 |  g = spruce
  0  0  4  1  1  3  0  4  0  3 |  h = quebrador
  2  0  0  0  0  0  0  0 15  0 |  i = arara
  0  2  6  0  6 15  3  1  0 55 |  j = caupuri
```

DL4j

=== Summary ===

Correctly Classified Instances 236 61.6188 %

Incorrectly Classified Instances 147 38.3812 %

Kappa statistic 0.5486

Mean absolute error 0.0967

Root mean squared error 0.2302

Relative absolute error 56.638 %

Root relative squared error 78.8426 %

Total Number of Instances 383

=== Detailed Accuracy By Class ===

TP Rate FP Rate Precision Recall F-Measure MCC ROC Area PRC Area Class

	0,944	0,016	0,739	0,944	0,829	0,827	0,986	0,803	hibrido
	0,840	0,022	0,724	0,840	0,778	0,763	0,977	0,804	pajezinho
	0,600	0,086	0,649	0,600	0,623	0,529	0,880	0,716	tucunaca
	0,938	0,011	0,789	0,938	0,857	0,854	0,937	0,926	cabrerana
	0,300	0,048	0,346	0,300	0,321	0,269	0,863	0,354	cabi
	0,476	0,084	0,526	0,476	0,500	0,408	0,843	0,523	ourinho
	0,600	0,028	0,643	0,600	0,621	0,590	0,942	0,747	spruce
	0,250	0,019	0,364	0,250	0,296	0,277	0,789	0,294	quebrador
	0,765	0,003	0,929	0,765	0,839	0,836	0,988	0,887	arara
	0,693	0,139	0,598	0,693	0,642	0,527	0,872	0,661	caupuri
Weighted Avg.	0,616	0,073	0,608	0,616	0,609	0,539	0,890	0,654	

=== Confusion Matrix ===

a	b	c	d	e	f	g	h	i	j	<-- classified as
17	0	0	0	0	0	0	0	1	0	a = hibrido
0	21	1	0	0	2	1	0	0	0	b = pajezinho
0	2	48	2	4	9	2	1	0	12	c = tucunaca
1	0	0	15	0	0	0	0	0	0	d = cabrerana
0	2	9	0	9	0	2	2	0	6	e = cabi
1	3	5	0	3	30	3	3	0	15	f = ourinho
1	0	0	0	3	1	18	0	0	7	g = spruce
0	0	6	1	2	2	0	4	0	1	h = quebrador
3	0	0	1	0	0	0	0	13	0	i = arara
0	1	5	0	5	13	2	1	0	61	j = caupuri

LEAF COMBINED SURFACES

RESELIB KNN

=== Summary ===

Correctly Classified Instances	526	68.4896 %
Incorrectly Classified Instances	242	31.5104 %
Kappa statistic	0.6296	
Mean absolute error	0.0756	
Root mean squared error	0.2344	
Relative absolute error	44.2965 %	
Root relative squared error	80.2857 %	
Total Number of Instances	768	

=== Detailed Accuracy By Class ===

	TP Rate	FP Rate	Precision	Recall	F-Measure	MCC	ROC Area	PRC Area	Class
	0,619	0,087	0,582	0,619	0,600	0,519	0,806	0,484	ourinho
	0,861	0,007	0,861	0,861	0,861	0,854	0,968	0,808	hibrido
	0,738	0,056	0,776	0,738	0,756	0,695	0,875	0,712	tucunaca
	0,790	0,025	0,731	0,790	0,760	0,738	0,908	0,646	spruce
	0,608	0,133	0,575	0,608	0,591	0,466	0,769	0,481	caupuri
	0,550	0,024	0,660	0,550	0,600	0,572	0,819	0,437	cabi
	0,780	0,022	0,709	0,780	0,743	0,725	0,958	0,721	pajezinho
	0,375	0,015	0,522	0,375	0,436	0,422	0,769	0,300	quebrador
	0,912	0,007	0,861	0,912	0,886	0,881	0,968	0,866	arara
	0,875	0,001	0,966	0,875	0,918	0,916	0,968	0,933	cabreana
Weighted Avg.	0,685	0,063	0,686	0,685	0,684	0,623	0,851	0,599	

=== Confusion Matrix ===

a	b	c	d	e	f	g	h	i	j	<-- classified as
78	0	7	5	25	4	5	2	0	0	a = ourinho
0	31	0	0	0	0	0	0	4	1	b = hibrido
9	0	118	1	26	2	3	1	0	0	c = tucunaca
2	0	1	49	7	2	0	1	0	0	d = spruce

27	0	15	9	107	9	4	5	0	0	e = caupuri
5	0	4	2	11	33	3	2	0	0	f = cabi
5	0	2	0	4	0	39	0	0	0	g = pajezinho
8	0	4	1	6	0	1	12	0	0	h = quebrador
0	3	0	0	0	0	0	0	31	0	i = arara
0	2	1	0	0	0	0	0	1	28	j = cabreana

LOCAL KNN

=== Summary ===

Correctly Classified Instances	532	69.2708 %
Incorrectly Classified Instances	236	30.7292 %
Kappa statistic	0.6396	
Mean absolute error	0.0682	
Root mean squared error	0.2307	
Relative absolute error	39.9299 %	
Root relative squared error	79.0097 %	
Total Number of Instances	768	

=== Detailed Accuracy By Class ===

TP Rate	FP Rate	Precision	Recall	F-Measure	MCC	ROC Area	PRC Area	Class
0,611	0,079	0,602	0,611	0,606	0,528	0,817	0,512	ourinho
0,917	0,010	0,825	0,917	0,868	0,863	0,954	0,776	hibrido
0,813	0,048	0,818	0,813	0,815	0,767	0,921	0,810	tucunaca
0,774	0,030	0,696	0,774	0,733	0,709	0,879	0,604	spruce
0,557	0,128	0,563	0,557	0,560	0,430	0,766	0,484	caupuri
0,550	0,037	0,559	0,550	0,555	0,517	0,796	0,376	cabi
0,780	0,014	0,796	0,780	0,788	0,773	0,904	0,730	pajezinho
0,469	0,018	0,536	0,469	0,500	0,481	0,751	0,299	quebrador
0,912	0,004	0,912	0,912	0,912	0,908	0,953	0,830	arara

	0,875	0,000	1,000	0,875	0,933	0,933	0,969	0,939	cabreana
Weighted Avg.	0,693	0,060	0,693	0,693	0,692	0,633	0,852	0,614	

=== Confusion Matrix ===

a	b	c	d	e	f	g	h	i	j	<-- classified as
77	0	6	4	27	6	4	2	0	0	| a = ourinho
0	33	0	0	0	0	0	0	3	0	| b = hibrido
8	0	130	0	17	2	1	2	0	0	| c = tucunaca
3	0	2	48	9	0	0	0	0	0	| d = spruce
27	0	12	16	98	14	2	7	0	0	| e = caupuri
4	0	4	1	14	33	2	2	0	0	| f = cabi
2	0	3	0	3	3	39	0	0	0	| g = pajezinho
7	0	2	0	6	1	1	15	0	0	| h = quebrador
0	3	0	0	0	0	0	0	31	0	| i = arara
0	4	0	0	0	0	0	0	0	28	| j = cabreana

OPTIMEZED FOREST

=== Summary ===

Correctly Classified Instances	521	67.8385 %
Incorrectly Classified Instances	247	32.1615 %
Kappa statistic	0.6171	
Mean absolute error	0.1093	
Root mean squared error	0.2161	
Relative absolute error	64.036 %	
Root relative squared error	74.0189 %	
Total Number of Instances	768	

=== Detailed Accuracy By Class ===

TP Rate FP Rate Precision Recall F-Measure MCC ROC Area PRC Area Class

0,548	0,065	0,622	0,548	0,582	0,508	0,905	0,673	ourinho
0,889	0,014	0,762	0,889	0,821	0,814	0,994	0,873	hibrido
0,756	0,076	0,725	0,756	0,740	0,670	0,938	0,850	tucunaca
0,661	0,021	0,732	0,661	0,695	0,671	0,934	0,812	spruce
0,750	0,179	0,555	0,750	0,638	0,519	0,886	0,679	caupuri
0,417	0,020	0,641	0,417	0,505	0,485	0,940	0,590	cabi
0,660	0,010	0,825	0,660	0,733	0,722	0,988	0,877	pajezinho
0,406	0,003	0,867	0,406	0,553	0,583	0,881	0,590	quebrador
0,794	0,004	0,900	0,794	0,844	0,839	0,996	0,942	arara
0,875	0,003	0,933	0,875	0,903	0,900	0,999	0,973	cabreana
Weighted Avg. 0,678	0,072	0,693	0,678	0,676	0,618	0,929	0,759	

=== Confusion Matrix ===

a	b	c	d	e	f	g	h	i	j	<-- classified as
69	0	11	5	36	4	1	0	0	0	a = ourinho
0	32	0	0	0	0	0	0	3	1	b = hibrido
11	0	121	1	23	2	1	1	0	0	c = tucunaca
3	0	5	41	13	0	0	0	0	0	d = spruce
19	0	11	5	132	6	2	1	0	0	e = caupuri
0	0	8	4	20	25	3	0	0	0	f = cabi
5	0	5	0	5	2	33	0	0	0	g = pajezinho
4	0	6	0	9	0	0	13	0	0	h = quebrador
0	6	0	0	0	0	0	0	27	1	i = arara
0	4	0	0	0	0	0	0	0	28	j = cabreana

RANDOM FOREST

=== Summary ===

Correctly Classified Instances 519 67.5781 %

Incorrectly Classified Instances 249 32.4219 %

Kappa statistic	0.6143
Mean absolute error	0.1094
Root mean squared error	0.216
Relative absolute error	64.0898 %
Root relative squared error	73.984 %
Total Number of Instances	768

=== Detailed Accuracy By Class ===

	TP Rate	FP Rate	Precision	Recall	F-Measure	MCC	ROC Area	PRC Area	Class
	0,548	0,065	0,622	0,548	0,582	0,508	0,904	0,673	ourinho
	0,889	0,012	0,780	0,889	0,831	0,824	0,994	0,862	hibrido
	0,756	0,081	0,712	0,756	0,733	0,661	0,937	0,845	tucunaca
	0,661	0,018	0,759	0,661	0,707	0,685	0,933	0,816	spruce
	0,722	0,179	0,545	0,722	0,621	0,496	0,885	0,679	caupuri
	0,433	0,020	0,650	0,433	0,520	0,499	0,942	0,601	cabi
	0,680	0,011	0,810	0,680	0,739	0,726	0,989	0,878	pajezinho
	0,406	0,004	0,813	0,406	0,542	0,563	0,892	0,585	quebrador
	0,824	0,004	0,903	0,824	0,862	0,856	0,996	0,942	arara
	0,875	0,003	0,933	0,875	0,903	0,900	0,999	0,974	cabreana
Weighted Avg.	0,676	0,073	0,689	0,676	0,674	0,614	0,929	0,759	

=== Confusion Matrix ===

a	b	c	d	e	f	g	h	i	j	<-- classified as
69	0	11	5	36	4	1	0	0	0	a = ourinho
0	32	0	0	0	0	0	0	3	1	b = hibrido
11	0	121	1	23	1	2	1	0	0	c = tucunaca
3	0	5	41	13	0	0	0	0	0	d = spruce
19	0	14	5	127	7	2	2	0	0	e = caupuri
1	0	8	2	20	26	3	0	0	0	f = cabi
4	0	5	0	5	2	34	0	0	0	g = pajezinho

4	0	6	0	9	0	0	13	0	0 |	h = quebrador
0	5	0	0	0	0	0	0	28	1 |	i = arara
0	4	0	0	0	0	0	0	0	28 |	j = cabreana

SVM

=== Summary ===

Correctly Classified Instances	535	69.6615 %
Incorrectly Classified Instances	233	30.3385 %
Kappa statistic	0.6424	
Mean absolute error	0.1627	
Root mean squared error	0.2769	
Relative absolute error	95.3186 %	
Root relative squared error	94.8188 %	
Total Number of Instances	768	

=== Detailed Accuracy By Class ===

	TP Rate	FP Rate	Precision	Recall	F-Measure	MCC	ROC Area	PRC Area	Class
	0,643	0,097	0,566	0,643	0,602	0,520	0,859	0,483	ourinho
	0,972	0,008	0,854	0,972	0,909	0,906	0,997	0,908	hibrido
	0,763	0,079	0,718	0,763	0,739	0,669	0,896	0,652	tucunaca
	0,742	0,020	0,767	0,742	0,754	0,733	0,916	0,673	spruce
	0,631	0,110	0,631	0,631	0,631	0,521	0,837	0,538	caupuri
	0,467	0,032	0,549	0,467	0,505	0,468	0,873	0,386	cabi
	0,840	0,007	0,894	0,840	0,866	0,857	0,992	0,864	pajezinho
	0,344	0,011	0,579	0,344	0,431	0,428	0,769	0,267	quebrador
	0,882	0,001	0,968	0,882	0,923	0,921	0,994	0,903	arara
	0,906	0,001	0,967	0,906	0,935	0,933	0,998	0,929	cabreana
Weighted Avg.	0,697	0,063	0,697	0,697	0,694	0,635	0,890	0,611	

=== Confusion Matrix ===

a	b	c	d	e	f	g	h	i	j	<-- classified as
81	0	7	3	25	6	2	2	0	0	a = ourinho
0	35	0	0	0	0	0	0	1	0	b = hibrido
15	0	122	1	16	3	2	1	0	0	c = tucunaca
4	0	2	46	7	2	0	1	0	0	d = spruce
27	0	17	7	111	11	0	3	0	0	e = caupuri
7	0	11	1	11	28	1	1	0	0	f = cabi
3	0	3	0	1	1	42	0	0	0	g = pajezinho
6	0	8	2	5	0	0	11	0	0	h = quebrador
0	3	0	0	0	0	0	0	30	1	i = arara
0	3	0	0	0	0	0	0	0	29	j = cabreana

DL4j

=== Summary ===

Correctly Classified Instances	495	64.4531 %
Incorrectly Classified Instances	273	35.5469 %
Kappa statistic	0.5827	
Mean absolute error	0.0847	
Root mean squared error	0.216	
Relative absolute error	49.6082 %	
Root relative squared error	73.967 %	
Total Number of Instances	768	

=== Detailed Accuracy By Class ===

TP Rate	FP Rate	Precision	Recall	F-Measure	MCC	ROC Area	PRC Area	Class
0,492	0,067	0,590	0,492	0,537	0,458	0,883	0,615	ourinho
0,917	0,011	0,805	0,917	0,857	0,852	0,996	0,868	hibrido
0,656	0,076	0,695	0,656	0,675	0,593	0,907	0,772	tucunaca

	0,694	0,031	0,662	0,694	0,677	0,648	0,946	0,808	spruce
	0,653	0,142	0,578	0,653	0,613	0,491	0,875	0,658	caupuri
	0,417	0,051	0,410	0,417	0,413	0,363	0,899	0,472	cabi
	0,840	0,022	0,724	0,840	0,778	0,763	0,985	0,916	pajezinho
	0,313	0,016	0,455	0,313	0,370	0,355	0,860	0,446	quebrador
	0,882	0,003	0,938	0,882	0,909	0,905	0,997	0,965	arara
	0,938	0,005	0,882	0,938	0,909	0,905	0,980	0,949	cabreana
Weighted Avg.	0,645	0,069	0,642	0,645	0,641	0,574	0,913	0,716	

=== Confusion Matrix ===

a	b	c	d	e	f	g	h	i	j	<-- classified as
62	1	8	5	34	8	6	2	0	0	a = ourinho
0	33	0	0	0	0	0	0	1	2	b = hibrido
11	0	105	3	19	11	6	4	0	1	c = tucunaca
1	0	1	43	10	4	2	1	0	0	d = spruce
21	1	16	9	115	11	0	2	1	0	e = caupuri
3	0	11	2	14	25	2	3	0	0	f = cabi
3	0	2	0	3	0	42	0	0	0	g = pajezinho
4	0	8	3	4	2	0	10	0	1	h = quebrador
0	4	0	0	0	0	0	0	30	0	i = arara
0	2	0	0	0	0	0	0	0	30	j = cabreana

RESULTS ANOVA AND TUKEY

ANOVA - Leaf adaxial surface

	Df	Sum Sq	Mean Sq	F value	Pr(>F)
Algoritmo	5	0.0067	0.001334	0.044	0.999
Residuals	54	1.6445	0.030454		

Tukey multiple comparisons of means

95% family-wise confidence level

Fit: aov(formula = F_Measure ~ Algoritmo, data = data_cima)

$Algoritmo

```
                                    diff        lwr       upr     p adj
Local KNN-DL4J                    0.0004 -0.2301791 0.2309791 1.0000000
Optimized Forest-DL4J            -0.0088 -0.2393791 0.2217791 0.9999973
Random Forest-DL4J               -0.0007 -0.2312791 0.2298791 1.0000000
Reselib KNN-DL4J                  0.0195 -0.2110791 0.2500791 0.9998595
SVM-DL4J                          0.0187 -0.2118791 0.2492791 0.9998857
Optimized Forest-Local KNN       -0.0092 -0.2397791 0.2213791 0.9999966
Random Forest-Local KNN          -0.0011 -0.2316791 0.2294791 1.0000000
Reselib KNN-Local KNN             0.0191 -0.2114791 0.2496791 0.9998731
SVM-Local KNN                     0.0183 -0.2122791 0.2488791 0.9998973
Random Forest-Optimized Forest    0.0081 -0.2224791 0.2386791 0.9999982
Reselib KNN-Optimized Forest      0.0283 -0.2022791 0.2588791 0.9991322
SVM-Optimized Forest              0.0275 -0.2030791 0.2580791 0.9992448
Reselib KNN-Random Forest         0.0202 -0.2103791 0.2507791 0.9998329
SVM-Random Forest                 0.0194 -0.2111791 0.2499791 0.9998630
SVM-Reselib KNN                  -0.0008 -0.2313791 0.2297791 1.0000000
```

ANOVA - Leaf abaxial surface

```
            Df Sum Sq  Mean Sq F value Pr(>F)
Algoritmo    5 0.0122 0.002431   0.083  0.995
Residuals   54 1.5734 0.029138
```

Tukey multiple comparisons of means

95% family-wise confidence level

Fit: aov(formula = F_Measure ~ Algoritmo, data = data_baixo)

$Algoritmo

```
                                    diff        lwr       upr     p adj
Local KNN-DL4J                    0.0415 -0.1840401 0.2670401 0.9940381
Optimized Forest-DL4J             0.0137 -0.2118401 0.2392401 0.9999726
```

Random Forest-DL4J 0.0103 -0.2152401 0.2358401 0.9999934

Reselib KNN-DL4J 0.0345 -0.1910401 0.2600401 0.9975013

SVM-DL4J 0.0233 -0.2022401 0.2488401 0.9996248

Optimized Forest-Local KNN -0.0278 -0.2533401 0.1977401 0.9991140

Random Forest-Local KNN -0.0312 -0.2567401 0.1943401 0.9984546

Reselib KNN-Local KNN -0.0070 -0.2325401 0.2185401 0.9999990

SVM-Local KNN -0.0182 -0.2437401 0.2073401 0.9998885

Random Forest-Optimized Forest -0.0034 -0.2289401 0.2221401 1.0000000

Reselib KNN-Optimized Forest 0.0208 -0.2047401 0.2463401 0.9997848

SVM-Optimized Forest 0.0096 -0.2159401 0.2351401 0.9999953

Reselib KNN-Random Forest 0.0242 -0.2013401 0.2497401 0.9995485

SVM-Random Forest 0.0130 -0.2125401 0.2385401 0.9999789

SVM-Reselib KNN -0.0112 -0.2367401 0.2143401 0.9999899

ANOVA - Leaf combined surfaces

Df Sum Sq Mean Sq F value Pr(>F)

Algoritmo 5 0.0068 0.001356 0.054 0.998

Residuals 54 1.3652 0.025282

Tukey multiple comparisons of means

95% family-wise confidence level

Fit: aov(formula = F_Measure ~ Algoritmo, data = data_todo)

$Algoritmo

diff lwr upr p adj

Local KNN-DL4J 0.0069 -0.2031898 0.2169898 0.9999987

Optimized Forest-DL4J -0.0187 -0.2287898 0.1913898 0.9998193

Random Forest-DL4J -0.0161 -0.2261898 0.1939898 0.9999136

Reselib KNN-DL4J -0.0050 -0.2150898 0.2050898 0.9999997

SVM-DL4J 0.0094 -0.2006898 0.2194898 0.9999940

Optimized Forest-Local KNN -0.0256 -0.2356898 0.1844898 0.9991619

Random Forest-Local KNN -0.0230 -0.2330898 0.1870898 0.9995020

Reselib KNN-Local KNN -0.0119 -0.2219898 0.1981898 0.9999807

SVM-Local KNN 0.0025 -0.2075898 0.2125898 1.0000000

Random Forest-Optimized Forest 0.0026 -0.2074898 0.2126898 1.0000000

Reselib KNN-Optimized Forest 0.0137 -0.1963898 0.2237898 0.9999611

SVM-Optimized Forest 0.0281 -0.1819898 0.2381898 0.9986857

Reselib KNN-Random Forest 0.0111 -0.1989898 0.2211898 0.9999863

SVM-Random Forest 0.0255 -0.1845898 0.2355898 0.9991777

SVM-Reselib KNN 0.0144 -0.1956898 0.2244898 0.9999502

ANOVA - Comparing leaf surfaces

Df Sum Sq Mean Sq F value Pr(>F)

Tipo 2 0.162 0.08122 3.119 0.0466 *

Residuals 177 4.609 0.02604

Signif. codes: 0 ‘***’ 0.001 ‘**’ 0.01 ‘*’ 0.05 ‘.’ 0.1 ‘ ’ 1

> print(TukeyHSD(anova_grupo))

Tukey multiple comparisons of means

95% family-wise confidence level

Fit: aov(formula = F_Measure ~ Tipo, data = dados_grupos)

$Tipo

diff lwr upr p adj

Leaf adaxial surface-Leaf abaxial surface 0.00270000 -0.066933938 0.07233394 0.9953802

Leaf combined surfaces-Leaf abaxial surface 0.06503333 -0.004600605 0.13466727 0.0726718

Leaf combined surfaces-Leaf adaxial surface 0.06233333 -0.007300605 0.13196727 0.0895292
